# Within-individual phenotypic plasticity in flowers fosters pollination niche shift

**DOI:** 10.1038/s41467-020-17875-1

**Published:** 2020-08-11

**Authors:** José M. Gómez, Francisco Perfectti, Cristina Armas, Eduardo Narbona, Adela González-Megías, Luis Navarro, Lucía DeSoto, Rubén Torices

**Affiliations:** 1 Estación Experimental de Zonas Áridas (EEZA-CSIC), Almería, Spain; 2 Research Unit Modeling Nature, Universidad de Granada, Granada, Spain; 3 Departamento de Genética, Universidad de Granada, Granada, Spain; 4 Departamento de Biología Molecular e Ingeniería Bioquímica, Universidad Pablo de Olavide, Sevilla, Spain; 5 Departamento de Zoología, Universidad de Granada, Granada, Spain; 6 Departamento de Biología Vegetal y Ciencias del Suelo, Universidad de Vigo, Vigo, Spain; 7 Departamento de Biología y Geología, Física y Química Inorgánica, Universidad Rey Juan Carlos, Móstoles, Spain

**Keywords:** Ecological networks, Evolutionary ecology, Natural variation in plants, Plant ecology, Plant evolution

## Abstract

Phenotypic plasticity, the ability of a genotype of producing different phenotypes when exposed to different environments, may impact ecological interactions. We study here how within-individual plasticity in *Moricandia arvensis* flowers modifies its pollination niche. During spring, this plant produces large, cross-shaped, UV-reflecting lilac flowers attracting mostly long-tongued large bees. However, unlike most co-occurring species, *M. arvensis* keeps flowering during the hot, dry summer due to its plasticity in key vegetative traits. Changes in temperature and photoperiod in summer trigger changes in gene expression and the production of small, rounded, UV-absorbing white flowers that attract a different assemblage of generalist pollinators. This shift in pollination niche potentially allows successful reproduction in harsh conditions, facilitating *M. arvensis* to face anthropogenic perturbations and climate change.

## Abstract (web summary)

Floral phenotypes impact interactions between plants and pollinators. Here, the authors show that *Moricandia arvensis* displays discrete seasonal plasticity in floral phenotype, with large, lilac flowers attracting long-tongued bees in spring and small, rounded, white flowers attracting generalist pollinators in summer.

## Introduction

The angiosperm flower is the quintessential example of an adaptive-integrated structure formed by multiple, functionally related parts that fit tightly together in a coordinated way to attract efficient pollinators, disseminate pollen, and promote plant reproduction^1,2^. Flowers may be plastic to attract certain pollinators or in response to some antagonists^3–5^. However, the environmental-induced modification of particular floral traits may imperil the correct functioning of the entire structure and diminish the fitness of the plastic phenotype^6^. In order to keep its functionality, the plasticity of flowers would thus require the concerted changes of all their parts and the production of a new fully integrated phenotype^1,7^. This abrupt multivariate plasticity is nevertheless unlikely, because the developmental requirements to produce any new complex structure constrain the ability to respond to immediate environmental changes^7,8^. Consequently, flowers tend to exhibit higher developmental canalization^5^ and express plasticity less frequently than other plant traits^4,5^.

Flowers have, in many cases, coevolved with pollinators^2,9^, resulting in a vast array of floral morphologies finely matching their behavioural and morphological traits^1,10^. Given the universal association between floral phenotype and pollinator diversity and identity, the environmental-driven modification of flowers may influence the preference and visitation rate of pollinators^3,11–14^. Therefore, floral plasticity will not only affect the performance of the plastic individuals but it may also reshape the structure and dynamics of their interaction networks and modify the identity and breadth of their pollination niches^15,16^. These ecological consequences of floral plasticity remain largely unexplored despite their great importance to understand how plants may potentially respond to changing environments^16^.

In this study, by combining ecological, physiological, and genetic approaches, we demonstrate both in the field and under controlled conditions the occurrence of abrupt multivariate within-individual floral plasticity in *Moricandia arvensis* that alter the structure of the pollinator network and cause a significant shift in pollination niches. Floral plasticity is expressed in this plant species at the level of the whole organ and as a consequence of the orchestrated response of multiple floral traits to the same environmental cues, causing the emergence of two radically different flowers in the same individual. This plasticity allows the same individual plants to exploit contrasting pollination niches by attracting different pollinators and reproduce successfully in a wide range of environments.

## Results and discussion

### Functional plasticity facilitates flowering in summer

**Fig. 1:**
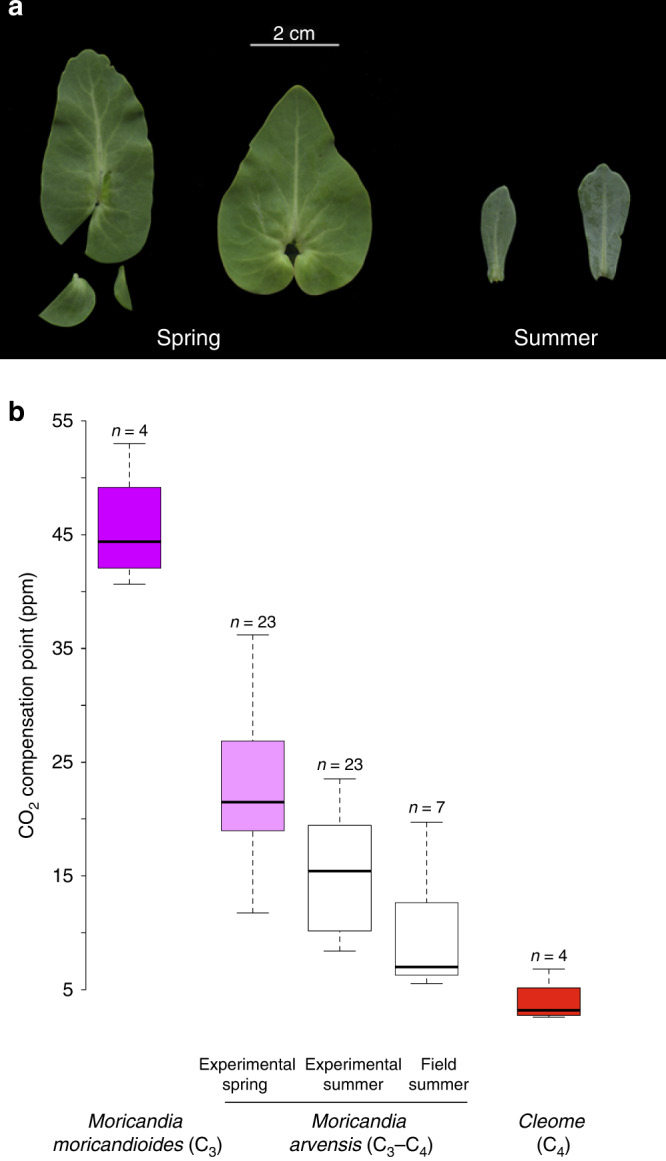
Plasticity in foliar and physiological traits. **a***Moricandia arvensis* leaves in spring and summer (leaves belonged to the same individual). **b** Box-plot showing the median, quartiles and interval confidences of the CO_2_ compensation points of the plants in spring (lilac box) and summer experimental conditions (the same plants replicated in each condition) and in summer in field conditions (white boxes). For comparison, we have added information from the C_3_ species *Moricandia moricandioides* (purple box, own data) and C_4_ species of the genus *Cleome* (red box), the closest C_4_ plants to *Moricandia* (see “Methods” section for details).

*M. arvensis* (Brassicaceae) is a perennial herb relative to cabbage and radish that inhabits dry semiarid and arid ecosystems of the Western Mediterranean. In these types of environments, *M. arvensis* faces two contrasting climatic conditions, mild and wet during spring but extremely dry and hot during summer (Supplementary Table 1). A general strategy to cope with stressful environmental conditions is to express plasticity in traits essential for physiological and ecological functions^4,17,18^. Accordingly, *M. arvensis* was plastic for functional traits associated with resource acquisition, producing denser and thicker leaves with more structural carbon and higher water use efficiency during summer than during spring (Fig. 1a, Supplementary Table 2, Supplementary Fig. 1; see supplementary material for detailed methods). The peculiar C_3_–C_4_ photosynthetic path of *M. arvensis*, a type of photosynthesis considered an intermediate step in the evolution from C_3_ to C_4_ photosynthesis^19^, may also help this species to face warm climates^4,20^. The CO_2_ compensation point of C_3_–C_4_ species lies between the values of C_3_ and C_4_ species^20^. However, we found that the *M. arvensis* CO_2_ compensation point shifted from typical C_3_–C_4_ values in spring conditions to values closer to C_4_ in summer conditions (Fig. 1b). Photosynthetic and vegetative plasticity may help to prolong the activity of *M. arvensis* into the hotter and drier season. Consequently, unlike most co-occurring species, *M. arvensis* extends its flowering well into summertime, starting to flower in early spring (late February to early March) and keeping blooming throughout the summer and even during autumn (Supplementary Table 1).

### Floral plasticity as a response to flowering in summer

**Fig. 2:**
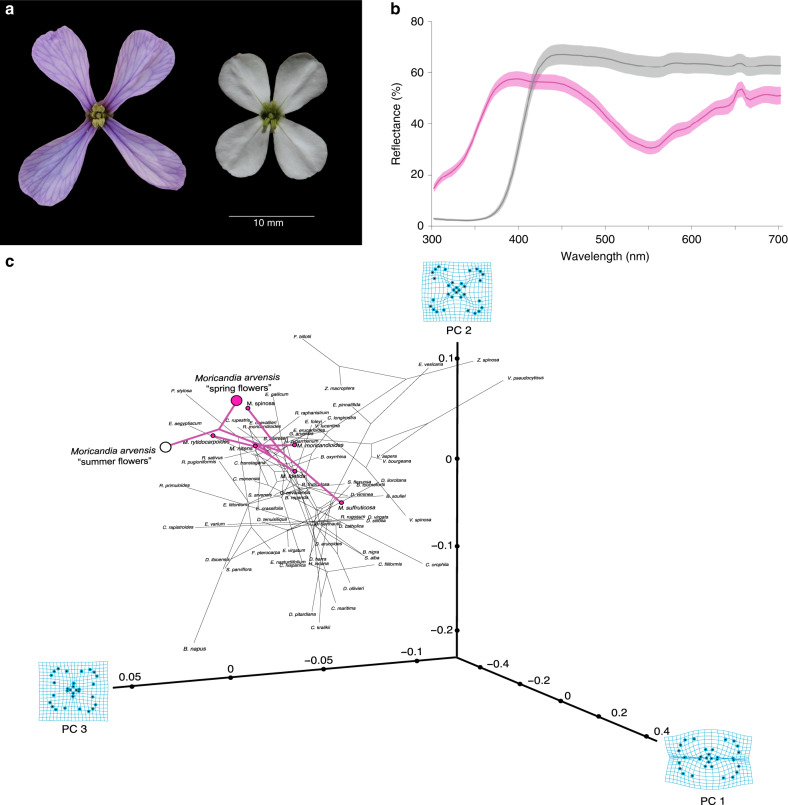
Within-individual changes in floral traits. **a** Floral phenotype of a spring (left) and a summer (right) flower belonging to the same individual. Pictures were taken to the same individual in March and June 2018, respectively, in Malaha population (Granada province, Spain). **b** Average UV–Vis spectral reflectance of petals of spring (lilac line) and summer (grey line) flowers. Shaded area represents standard errors (*n* = 10 spring flowers and 10 summer flowers). **c** Phylomorphospace projection of the first three principal components of the corolla shape onto the phylogeny of the Brassiceae tribe (*n* = 72 species). It is shown the change in shape as deformation grids associated with extreme values of each component. The *M. arvensis* spring flower is shown as a large lilac dot and the *M. arvensis* summer flower as a large white dot. The *Moricandia* clade is shown in lilac.

**Fig. 3:**
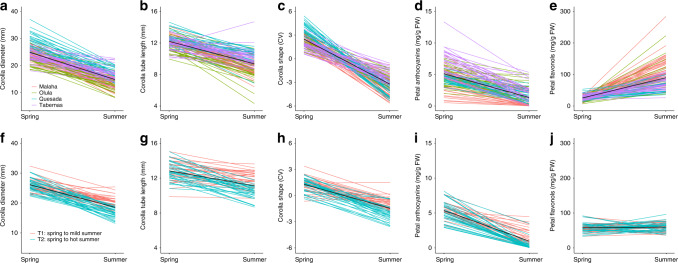
Within-individual reaction norms of floral traits in field and experimental conditions. The upper five panels refer to field conditions, pooling the individuals of the four studied populations (*n* = 117 individuals). The lower five panels refer to experimental conditions, pooling individuals of the two treatments (*n* = 58 individuals). **a**, **f** Corolla diameter, in mm. **b**, **g** Corolla tube length, in mm. **c**, **h** Corolla shape, as the value of the CV component of a landmark-base geometric morphometric analysis. **d**, **i** Anthocyanin concentration in corolla, expressed as cyanidin-3-glucoside equivalents per fresh weight. **e**, **j** UV-absorbing flavonols concentration in corolla, expressed kaempferol-3-glucoside equivalents per fresh weight.

By flowering in two contrasting environmental conditions, the same individuals produced flowers differing radically in phenotype across seasons (Fig. 2a). During spring, plants produced large, cross-shaped, bright lilac UV-reflecting flowers (Fig. 2a, b), similar to the archetypal *Moricandia* flower^21–23^. During summer, in contrast, plants produced small, rounded, UV-absorbing white flowers (Fig. 2a, b) resembling the flowers of species belonging to different clades of the genus or even to different genera and taxonomic tribes in the Brassicaceae family (Fig. 2c, Supplementary Table 3)^21–23^. The colour of the flower during spring was caused in *M. arvensis* by the accumulation of anthocyanin derivatives (Supplementary Table 4), although other flavonoids absorbing in the UV range, such as the flavonols quercetin, kaempferol, and isorhamnetin, were also present in the petals (Supplementary Table 4). As a consequence of this change in phenotype, individuals exhibited significant within-individual plasticity for all floral traits: corolla diameter, tube length, shape, and anthocyanin and flavonol concentration (Fig. 3a–e, Supplementary Table 5). This plasticity in floral traits was a widespread phenomenon since any individual from any of the studied populations that happened to flower during summer expressed it (Fig. 3, Supplementary Tables 5 and 6). This seasonal phenotypic change in the *M. arvensis* flower was coordinated across floral traits, since there were significant correlations between the plasticity’s magnitudes of corolla diameter, tube length, shape, and flavonols (Supplementary Table 7). Consequently, both types of flowers were similarly integrated (8.5 ± 0.9% vs. 14.6 ± 3.3%; Wilcoxon rank test = 3.0, *p* = 0.2; *N* = 4 populations, 5 traits, 117 individuals) (see supplementary material for detailed methods). Likewise, both types of flowers were fully functional and contributed to the overall reproductive success of the plants, since they produced a similar amount of seeds in field conditions (49.2 ± 2.1 vs. 56.7 ± 1.9 seeds/flower, respectively, *N* = 4 populations, 117 individuals; Supplementary Table 8). Finally, both types of flowers required pollen vectors for full seed set, since flowers excluded from pollinators and experimentally self-fertilized did not produce seeds (Supplementary Fig. 2).

The floral plasticity exhibited by *M. arvensis* in the field was triggered by changes in temperature and photoperiod, since it was reproduced in controlled conditions by modifying these two environmental factors (Fig. 3f–j) (see supplementary material for detailed methods). All floral traits, except petal flavonols, were plastic when plants growing in experimental spring conditions were afterward exposed to mild summer or hot summer conditions (Fig. 3f–j, Supplementary Table 9). There was an increase in the magnitude of plasticity with the severity of the experimental summer conditions. The individual slopes were lower in mild summer conditions than in hot summer conditions, and the reaction norms of plants from the hot summer condition were always below the reaction norms of the mild summer condition (Fig. 3f–j, Supplementary Table 9). Control treatment (plants submitted to two rounds of spring conditions) did not elicit floral plasticity (Supplementary Table 9), indicating that these changes in floral traits were a consequence of changes in the experimental abiotic conditions rather than an artefact of the time that the plants were in chambers. Floral plasticity was, nevertheless, stronger in field conditions than in experimental conditions (Supplementary Fig. 3), suggesting that although temperature and photoperiod prompt floral plasticity in *M. arvensis*, other environmental factors are also involved in the change.

We checked whether the change in *M. arvensis* floral phenotype, rather than being a plastic response to environmental conditions, was a consequence of the whole-plant ontogenetic changes (i.e., heteroblasty in plants)^24,25^ by quantifying, both observationally and experimentally, the potential reversibility of floral phenotype (see supplementary material for detailed methods). All floral traits were reversible in both natural and experimental plants, and the flowers produced after summer, when facing again milder conditions, resembled the spring flowers (Supplementary Fig. 4, Supplementary Tables 10 and 11). This outcome shows that this within-individual change in floral phenotype is indeed a consequence of phenotypic plasticity rather than a consequence of ontogeny.

Altogether, our findings demonstrate that *M. arvensis* produces two types of functional, well-integrated, animal-pollinated flowers. Floral plasticity usually expresses as subtle quantitative changes, such as variations in flower or petal size, colour, nectar production, floral volatiles, and number of reproductive structures^3,12–14,26,27^ or changes in the proportion of self-pollinating cleistogamous flowers^28^. In contrast, such an extreme multivariate within-individual floral plasticity as the one reported here, stronger even than plasticity in key functional foliar traits (Supplementary Fig. 3) and causing the appearance of two sets of animal-pollinated flowers that radically differ in their phenotype, has not been previously found^3^.

### Floral gene expression changes in summer conditions

**Fig. 4:**
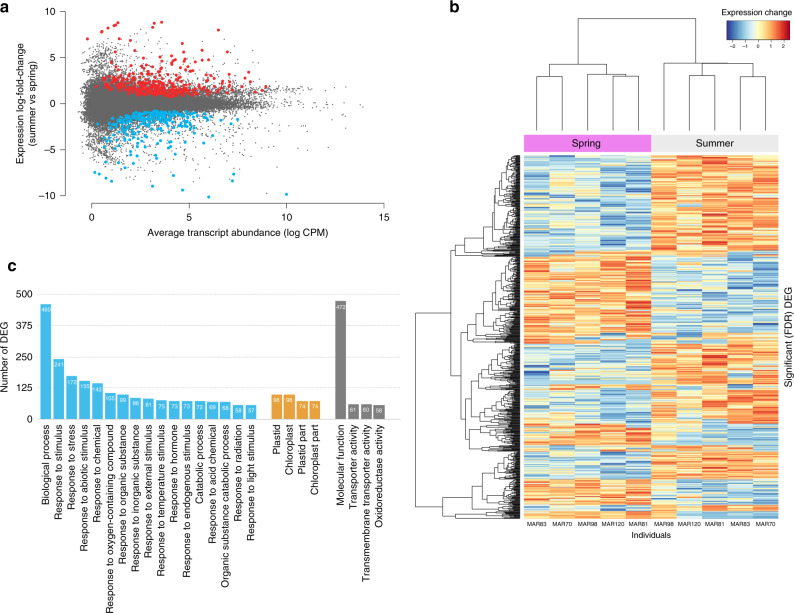
Between-season differences in floral transcriptomes. **a** MA plot showing the change in expression levels, quantified as log-fold change ((expression level in summer flowers−expression level in spring flowers)/expression level in spring flowers) in samples from one starting-to-open flower bud during experimental spring conditions, and another flower bud during experimental mild summer conditions in five plants. Each dot represents a transcript, and log_2_-fold change (summer flower/spring flower) is plotted against average abundance in log of counts per million (log CPM). Red dots indicate differentially overexpressed transcripts (false discovery rate adjusted *P*-values < 0.05) in summer flowers, blue dots indicate differentially under-expressed transcripts in summer flowers. **b** Heatmap of hierarchical clustering of the expression matrix of the 627 differentially expressed genes (DEG) in the five individuals in each season. **c** The first 25 enriched gene ontology (GO) classes after the comparison of GO terms between the DEG and the 47,440 genes that passed the criteria for inclusion in the analyses. GO terms are distributed in three main categories: biological processes (blue bars), cellular components (orange bars), and molecular functions (grey bars). Numbers inside columns indicate the number of DEG in that category.

We analysed the floral transcriptomes of five experimental individuals consecutively exposed to spring and summer conditions (Supplementary Table 12; see “Methods” section). Overall gene expression was different between these two experimental conditions, with 256 genes expressed significantly more in spring conditions and 371 in summer (Fig. 4a, b). These differentially expressed genes (DEG) were enriched in GO terms related to responses to stress, temperature, radiation, light, and other environmental stimuli (Fig. 4c; Supplementary Data 1). In particular, summer flowers showed higher expression of genes coding for heat shock proteins (Fig. 4c; Supplementary Data 1). The higher expression of genes encoding heat shock proteins could help to maintain other proteins’ functionality in summer^29^. Heat shock proteins, particularly Hsp90 and their co-chaperones, have also been associated with the perception and transduction of signals related to programmed plasticity, that is, the directional shift in phenotype in response to environmental changes^30^.

Not only buffering genes changed their expression in the experimental flowers, but genes potentially associated with the observed phenotypic changes in size, shape, and colour did. For example, the *M. arvensis* homologous of RADIALIS, a transcription factor associated with flower asymmetry in *Antirrhinum majus*, was expressed more in summer flowers (Supplementary Data 2). Likewise, several homologous to *Arabidopsis thaliana* genes associated with growth-promoting processes, such as *EXO*, related to cell expansion, *SAUR*10, related to cell elongation, *ARF*5 and *ARF*8, involved in auxin-response, and *BON*2, related to cell death, were differentially expressed in spring and summer flowers of *M. arvensis* (Supplementary Data 2). Finally, there was a reduction in the expression of several genes of the anthocyanin biosynthetic pathway, including both structural genes such as *PAL*, 4*CL*, *CHS*, *DFR*, and several UDP-glucosyl transferases (*U*78*D*2 and *U*75*C*1), as well as transcriptional factors such as *MYB*90 and *TTG*1 (Supplementary Data 2, Supplementary Fig. 5). These changes in structural and regulatory genes suggest a coordinated response that could potentially drive flower colour changes in response to high temperatures and long photoperiods^31^.

### Floral plasticity affects the interaction with pollinators

**Fig. 5:**
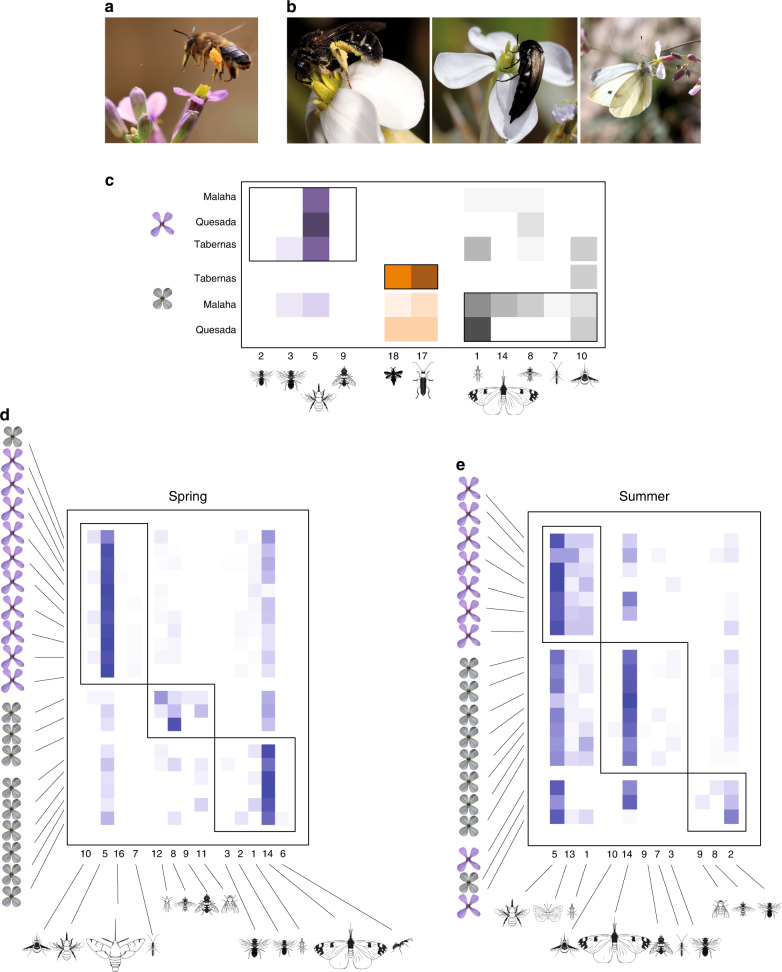
Seasonal changes in pollination niche. **a** Main pollinator of spring flowers: long-tongued bee (*Anthophora* sp.). **b** Main pollinators of summer flowers: small bee (*Lasioglossum* sp.), small beetle (*Mordellistena* sp.) and butterfly (*Pieris* sp.) (photographs taken by F. Perfectti). Outcome of the modularity analysis of the pollination networks built upon the visitation rate of the main pollinator functional groups **c** to three *M. arvensis* populations during summer and spring; **d** to experimental plants displaying spring flowers and summer flowers during spring in the field; **e** to experimental plants displaying spring flowers and summer flowers during summer in the field. Pollinator Functional Groups: 1. short-tongued small bees; 2. short-tongued medium-sized bees; 3. short-tongued large bees; 5. long-tongued large bees; 6. ants; 7. small wasps; 8. small hoverflies; 9. large hoverflies; 10. beeflies; 11. large flies; 12. long-tongued small flies; 13. small butterflies; 14. large butterflies; 16. hawkmoths; 17. beetles; 18. trips. Insect silhouettes drawn by Divulgare (www.divulgare.net) under a Creative Common license (http://creativecommons.org/licenses/by-nc-sa/3.0).

The display of two different animal-pollinated flowers during spring and summer caused the same *M. arvensis* plant to attract different pollinators with different preferences and effectiveness. *M. arvensis* flowers were visited mostly by long-tongued large bees in spring (Fig. 5a), but mostly by short-tongued small bees, butterflies, and beetles in summer (Fig. 5b, Supplementary Table 13). We explored whether this change in pollinators results in a pollination niche shift by determining the modularity of the network describing the frequency of visits of main pollinator functional groups to each studied population^32–34^ (see supplementary material for detailed methods). This pollinator network was significantly modular (modularity = 0.46, *p* < 0.0001), suggesting the occurrence of several pollination niches in *M. arvensis* (Fig. 5c). Most important, all studied plant populations changed between modules from spring to summer (Fig. 5c), indicating that they changed seasonally between pollination niches. By displaying two contrasting flowers, the same plant is attracting two different pollinator assemblages and exploits two different pollination niches across seasons.

To assess how profound was this seasonal change in the pollination niche, we determined their position within the niche space of those plant species phylogenetically related to *M. arvensis* (Supplementary Data 3–6). This network was again significantly modular (modularity = 0.42, *p* < 0.0001), with the niche space divided into five different pollination niches (Supplementary Fig. 6). Interestingly, during spring, when *M. arvensis* plants display flowers similar to other *Moricandia* species^21,22^, *M. arvensis* belongs to the canonical *Moricandia* pollination niche dominated by long-tongued large bees (Supplementary Fig. 6). During summer, by displaying a completely different flower, *M. arvensis* jumps to a contrasting and more diverse pollination niche, including short-tongued bees, beetles, and butterflies. This new niche is shared with only one species of *Moricandia*, *M. foetida*, which displays small rounded whitish flowers^23^, like *M. arvensis* during summer. This finding suggests that the pollination niche shift between *M. arvensis* spring and summer flower is severe.

The diversity and composition of the pollinator fauna change seasonally in the Mediterranean area^35^. Accordingly, the observed change in pollination niches could be a consequence of *M. arvensis* flowers being visited by the pollinators available each season. To know whether the exploitation of this new pollination niche was just a mere response to the seasonal replacement in floral visitors or was a real consequence of floral plasticity, we experimentally offered spring and summer flowers to pollinators, both during spring and summer (see supplementary material for detailed methods). During spring, experimental spring flowers received more visits than experimental summer flowers (1.7 ± 0.2 vs. 0.6 ± 0.1 visits flower^−1^ h^−1^, respectively, mean ± 1. s.e.m, *F* = 46.7, df = 1,18, *p* < 0.0001). During summer, both types of experimental flowers received similar number of visits (2.6 ± 0.5 vs. 2.7 ± 0.3 visits flower^−1^ h^−1^, respectively; *F* = 0.07, df = 1, 25, *p* = 0.80, ANOVA). The experimental networks were modular in both seasons (spring modularity = 0.33, summer modularity = 0.18, all *p* < 0.0001), indicating that the two floral phenotypes were visited by different pollinator faunas and thereby belonged to different niches (Fig. 5d, e). Experimental spring flowers belonged to the same pollination niche, associated with long-tongued large bees, in both spring and summer seasons (Fig. 5d, e). In contrast, most experimental summer flowers belonged to pollination niches dominated by butterflies and other generalist pollinators in both seasons (Fig. 5d, e). These experiments indicate that the observed shift in pollination niche was due to the change in floral phenotype rather than a consequence of a change in the availability of different pollinators in each season. In brief, our study shows that the discrete within-individual floral plasticity allows *M. arvensis* to jump to a different region of the floral phenotypic space and exploit alternative pollination niches.

### Is floral plasticity an adaptation to novel environments?

Phenotypic plasticity impacts the ecological interactions of many organisms^11,12,14,36^. In many cases, this plasticity is adaptive, with the expression of the plastic traits finely adjusted to the preference, abundance, or behaviour of the interacting partners^14^. Accordingly, the floral plasticity of *M. arvensis* could be an adaptation to the summer pollination environment. The rampant presence of significant G × E interactions (Supplementary Tables 2, 5 and 8) indicates that individuals differ in the magnitude of floral plasticity, suggesting that natural selection can operate on *M. arvensis’* floral plasticity. Our pollination experiments show an association between floral phenotypes and pollinator types, a pattern widely accepted as circumstantial evidence of adaptation to pollinators^10,37,38^. However, this association can also occur not only by plants adapting to pollinators but also by pollinators associating with their preferred floral phenotype^39,40^. Therefore, with the evidence at hand, it is premature to reach firm conclusions. We think that in *M. arvensis*, the shift in pollination niche is probably a consequence rather than a cause of the observed floral plasticity. Under this idea, the vegetative and photosynthetic plasticity may have allowed *M. arvensis* to function and even flower in summer. Subsequent genetic responses to summer temperature and photoperiod may have promoted the coordinated change of floral traits and the appearance of a new floral phenotype. This floral phenotype, utterly different to the one produced during springtime but fully integrated and functional, was attractive to a new set of pollinators, causing the plant to expand its pollination niche during summer.

### Consequences of plasticity-mediated niche shifts

Phenotypic plasticity may have important consequences for *M. arvensis*. By being able to flower and attract efficient pollinators even in summer, this species produced viable seeds during this harsh season, when most other co-occurring plants are inactive and dormant. Plasticity is thus beneficial itself since it provides the plastic phenotype with an extra amount of seeds. Furthermore, floral plasticity and the subsequent shift in the pollination niche allow *M. arvensis* to reproduce successfully in a broader range of environments, a property facilitating the colonization of new territories^15,16^ and the ability to withstand the adverse effects of global change^41,42^. This may explain the rapid expansion of *M. arvensis* geographic range in historical times^22,23,41^. Our study suggests that the effect of phenotypic plasticity in the structure and dynamics of biotic niches and ecological networks may help plants to respond to present-day anthropogenic perturbations and future climate change scenarios.

## Methods

### Field sampling design

To determine if there was within-individual plasticity in floral traits between spring and summer conditions, 50 plants of each of four populations from SE Spain (Supplementary Table 1) were marked at the onset of the flowering period in late February–early March 2018. The phenotype of two flowers per individual was quantified (see below). We revisited each population during summer (June 2018) and the same floral traits were quantified in the summer flowers of those plants still flowering (117 plants; Supplementary Table 1).

### Experimental design

We performed an experiment testing the effect of temperature and photoperiod in floral plasticity. It included three treatments: (1) Treatment 1, where 30 plants flowered first in conditions mimicking the spring temperature and photoperiod of Mediterranean Spain (day/night = 10/14 h, temperature = 20/10 °C, average daily temperature = 14.2 °C; see Supplementary Table 1), and afterwards in conditions mimicking a mild summer (day/night = 16/8 h, temperature = 30/20 °C, average daily temperature = 23.8 °C). (2) Treatment 2, where 30 plants flowered first in spring conditions and afterwards in hot summer conditions (day/night = 16/8 h, temperature = 35/25 °C, average daily temperature = 28.8 °C). (3) Treatment 3 (control) where 15 plants flowered first in spring conditions, and afterwards they flowered again in spring conditions. For all treatments, we removed flowers before starting the second round of flowering.

We experimentally tested the occurrence of reverse plasticity by performing a Treatment 4 in which 15 plants from Treatment 1 that flowered both during spring and summer conditions were again submitted to a period mimicking spring conditions (Supplementary Table 1).

### Floral traits

We measured, both in field and experimental conditions, three floral traits during spring (or under experimental spring conditions) and in summer (or under experimental hot summer conditions). These traits were corolla size, corolla shape and corolla colour.

Corolla size of each studied flower was estimated by means of two traits: (1) corolla diameter, estimated as the distance in mm between the edge of two opposite petals. (2) Corolla tube length, the distance in mm between the corolla tube aperture and the base of the sepals. These variables were measured by using a digital calliper with ±0.1 mm of error.

Corolla shape variation was studied using geometric morphometric tools based on a landmark-based methodology^43^. For this, in each of the two selected flowers per individual plant studied in each of the four populations, we took a digital photo of the front view and planar position. We defined 32 co-planar landmarks covering the corolla shape and using midrib, primary and secondary veins and petal extremes and connections^21,44^. From the two-dimensional coordinates of landmarks, we extracted shape information and computed the generalized orthogonal least-squares Procrustes averages using the generalized procrustes analysis (GPA) superposition method. Due to the intrinsic symmetry pattern exhibited by Brassicaceae flowers, we did the analyses considering both the symmetric and asymmetric components of the shape^45–47^. We performed a principal component analysis (PCA) on the GPA-aligned specimens, and afterwards, we did a canonical variate analysis (CVA) to explore the difference in shape between season and populations^43,47^. Geometric morphometric analyses were performed in the R packages ‘geomorph’^48^, ‘Morpho’^47^ and ‘shapes’^49,50^.

To explore the relative position of the corolla shape of spring and summer flowers in the morphospace created by the species most related phylogenetically with *M. arvensis*, we performed a phylomorphospace. This analysis creates a plot of the main principal dimensions (the three first principal components in this case) of a tangent space for the Procrustes shape variables of the pool of species considered in the analysis and superimposed the phylogenetic tree relating this species in this plot^51,52^. By doing this, this analysis reveals how the shape evolves. To perform this analysis, we collected information on the corolla shape of 72 additional species belonging to the Brassicaceae tribe Brassiceae, the tribe to which *M. arvensis* belongs (Supplementary Table 3). We followed the same procedure as with *M. arvensis*, using the same number of landmarks and computing the generalized orthogonal least-squares Procrustes averages using GPA superposition method. In this analysis, we kept separate the spring and summer flowers of *M. arvensis*. The phylogenetic relationship between these 72 species was obtained by making a supertree using Brassicaceae trees hosted in the repository TreeBASE Web (TreeBase.org)^53^. We first downloaded individual phylogenetic trees from TreeBASE. Second, we concatenated all these individual trees and made a skeleton supertree. Finally, we pruned this supertree, keeping only the species included in the geometric morphometric analysis, and insert the two ‘pseudospecies’ of *M. arvensis* (spring and summer) as sister species. Afterwards, we projected the value of the three first components of each species on a 3D phylogenetically explicit plot. The phylogenetic analysis was performed in the R packages ‘treeman’^54^, ‘phangorn’^55^, ‘phytools’^56^ and ‘treebase’^53^, whereas the phylomorphospace analysis was performed in the R packages ‘geomorph’^48^.

The corolla colour of *M. arvensis* is produced by the accumulation of flavonoids^57,58^. Anthocyanin and non-anthocyanin flavonoids present in the petals of *M. arvensis* were analysed by ultra-performance liquid chromatography (UPLC) (ACQUITY System I-Class, Waters) coupled with quadrupole time-of-flight mass spectrometry (SYNAPT G2 HDMS Q-TOF, Waters). Analytical separation of flavonoids was performed on an Acquity HSST33 analytical column (150 mm × 2.1 mm internal diameter, 1.8 μm). A mobile phase with a gradient programme combining deionized water with 0.5% of acetic acid as solvent A and acetonitrile with 0.5% of acetic acid as solvent B was used. The initial conditions were 95% A and 5% B and a linear gradient was then established to reach 95% (v/v) of B. The total run time was 15 min and the post-delay time was 5 min. The mobile phase flow rate was 0.4 mL min^−1^. After chromatographic separation, a high-resolution mass spectrometry analysis was carried out in positive electrospray ionization (ESI+). The ionization source parameters using high-purity nitrogen were set at 600 L h^−1^ for desolvation gas flow and 30 L h^−1^ for cone gas flow. Spectra were recorded over the mass/charge (*m*/*z*) range of 50–1500. Data were recorded and processed using MassLynx software. The flavonoids present in the petal extracts were characterized according to their retention times, mass spectra and molecular formula, and compared with published data when available. We calculated the relative abundance of each compound in both lilac and white petal samples (*N* = 5 and 2, respectively) using peak intensities.

Quantification of flavonoids present in flowers of *M. arvensis* was performed spectrophotometrically. Two flowers of each plant used in field and experimental studies were analysed in each blooming period. We collected the four petals of a flower. Flavonoids were extracted in 1.5 ml of MeOH:HCl (99:1% v-v) and stored at −80 °C in the dark, following the procedure described in ref. ^34^. Two replicas of 200 μL for each sample were measured in a Multiskan GO microplate spectrophotometer (Thermo Fisher Scientific Inc., MA, USA). Main flavonoid classes present in the petals of *M. arvensis* are anthocyanins (cyanidin derivatives) and flavonols (kaempferol, quercetin and isorhamnetin derivatives; Supplementary Table 4)^57,58^. Thus, total anthocyanins and flavonols were quantified as absorbance at 520 and 350 nm, respectively. Their concentrations were calculated using five-point calibration curves of cyanidin-3-glucoside chloride (Sigma-Aldrich, Steinheim, Germany) and kaempferol-3-glucoside standards (Extrasynthese, Genay, France) and expressed as cyanidin-3-glucoside and kaempferol-3-glucoside equivalents in fresh weight (mg g^−1^ FW), respectively.

Objective quantification of petal colour of lilac and white petals of *M. arvensis* was performed by measuring their UV–Vis spectral reflectance. A petal of a flower of each colour morph (*N* = 10) were measured with a Jaz portable spectrometer (Ocean Optics Inc., Dunedin, FL, USA) equipped with a deuterium–tungsten halogen light source (200–2000 nm) and a black metal probe holder (6 mm diameter opening at 45°). Reflectance, relative to a white standard (WS-1-SL), was analysed with SpectraSuite v.10.7.1 software (Ocean Optics). To maximize the amount of light used in reflectance measurements and to reduce occasionally erratic reflectance values at individual nm, we set an integration time of 2 s and smoothing boxcar width of 12, respectively^59^.

### Foliar traits

We measured, both in field and experimental conditions, five leaf traits during spring (or under experimental spring conditions) and in summer (or under experimental hot summer conditions). These traits were the specific leaf area (SLA, m^2^ kg^−1^), the leaf dry matter content (LDMC, mg g^−1^), the carbon-to-nitrogen content of leaves (C:N ratio), the isotopic signature of ^13^C in leaves (δ^13^C, ‰), and the CO_2_ compensation point and the slope of the *A*–*C*_i_ curve.

SLA and LDMC were measured following standard protocols^60^. For SLA and LDMC we collected three fully expanded and mature leaves without any visible damage (e.g., herbivory, pathogen attack) from the base, midsection and apical part of outer stems (that is, leaves were not shaded by other leaves) and at random aspects. Leaves were rehydrated overnight in the dark and subsequently weighted and scanned. Leaf area was measured using the Midebmp software (Almería, Spain). Leaves were dried in the oven at 60 °C and weighted after 72 h. From these measurements, we calculated the SLA as the one-sided area of the fully rehydrated fresh leaf divided by its dry mass, while the LDMC is the ratio between the leaf dry mass and the fully rehydrated fresh mass.

Carbon isotopic signature (δ^13^C), as well as the C and N relative content in leaves, were analysed in a couple of fully expanded leaves per plant without any visible damage. Oven-dry leaves were ground in a ball mill MM400 (Retsch GmbH, Haan, Germany) at 3000 rpm for 1 min to obtain a fine powder, which was stored in Eppendorf tubes. We wrapped 0.003 g of each sample in tin capsules D1008 (Elemental Microanalysis, United Kingdom). Leaf δ^13^C and leaf C and N relative content (in mass percentage) were determined at the Stable Isotope Analysis Lab—Centro de Instrumentación Científica (CIC) of the University of Granada (Spain) with a GC IsoLink—MS—Delta V continuous flow mass spectrometer (MS) system that includes a ISQ-QD single quadrupole MS and a gas chromatographer Trace 1310 (Thermo Fisher Scientific™, Spain). The isotopic abundance was expressed in parts per thousand (‰) as

1 $$\delta = \left( {{R}_{{\mathrm{sample}}}/{R}_{{\mathrm{standard}}}-1} \right) \times 1000$$

where *R*_sample_ and *R*_standard_ are the molar ratios of heavy (^13^C) to light (^12^C) stable isotopes of the sample (*R*_sample_) and an international standard (*R*_standard_). MS precision was 0.15‰ for carbon, based on replicate analyses of standard reference materials.

We measured responses of CO_2_ assimilation rate (*A*) versus calculated substomatal or intercellular CO_2_ concentration (*C*_i_) (henceforth, *A*–*C*_i_ curves) to determine the instantaneous photosynthetic metabolism of plants of the intermediate C_3_–C_4_ species *M. arvensis* on plants grown under the two experimental conditions (*N*= 22 plants, spring and hot summer conditions). Gas exchange measurements were performed on one to two mature, fully expanded leaves per plant and experimental condition using a LICOR 6400 (LI-COR Biosciences, Lincoln, USA) and following the standard recommendations to correct leakage errors^61–63^. Cuvette conditions were maintained at a constant photosynthetic photon flux density (PPFD) of 1500 µmol m^−2^ s^−1^, a vapour pressure deficit (VPD) that ranged from 1.0 to <2.4 kPa and a cuvette temperature that was either 20 or 30 °C (see below). *A*–*C*_i_ curves were measured at a series of ambient (i.e., reference) CO_2_ concentrations (*C*_a_) starting at 400 ppm. The *C*_a_ was then lowered stepwise from 400 to 40 ppm and then increased again to 1600 ppm, all on 21 steps: 400, 300, 200, 150, 100, 80, 60, 50, 40, 40, 40, 60, 100, 200, 400, 600, 800, 1000, 1200, 1400, 1600 ppm of CO_2_.

The CO_2_ compensation point (substomatal CO_2_ concentration value at which *A* is zero) and initial slope of each *A*–*C*_i_ curve were calculated performing a linear regression of 10 different *C*_a_ values (all below 300 ppm). *C*_a_ instead of *C*_i_ values were used in the calculations as we had some technical issues with the LICOR in one of the two measurement phases. This fact might overestimate absolute results for CO_2_ compensation points, but the relative differences (if any) between the two experimental conditions (spring and summer) remain. Measurements of gas exchange were made at two different LICOR cuvette temperatures (20 and 30 °C) for plants growing in spring and hot summer experimental conditions, respectively. Although the CO_2_ compensation point can increase with cuvette temperature^61^, we measured the compensation points of six plants grown in spring chamber conditions and at these two contrasting cuvette temperatures, and results did not differ (CO_2_ compensation point ± s.e.m.: 29.72 ± 4.03 vs. 31.42 ± 4.03 ppm at 20 and 30 °C bulk temperature of the LICOR chamber, respectively; GLM results, *F*_1,4_ = 0.09; *P*-value = 0.7796; *N*= 6 individuals). Moreover, mean CO_2_ compensation points of plants grown in spring chamber conditions were significantly higher (not lower) than values for the same plants grown in hot-summer conditions (see main text; mean ± s.e.m.: 25.0 ± 0.21 ppm in spring versus 14.7 ± 0.16 ppm in hot summer conditions).

To compare the CO_2_ compensation point of *M. arvensis* with pure C_3_ and C_4_ species, we also calculated the CO_2_ compensation point for four individuals of the C_3_ species *Moricandia moricandioides*. In addition, we have compiled from the literature information on the CO_2_ compensation point of several C_4_ species belonging to the genus *Cleome*^64–66^. This genus is the closest C_4_ plant to *Moricandia*^67^.

### Reproduction and mating system

We collected 10 ripe fruits belonging to spring flowers and 10 ripe fruits belonging to summer flowers from each of the studied plants. These fruits were taken to the lab, where we determined under magnifying glasses (×60) the total number of ovules produced per flower and the number of ripe seeds per fruit^68^^.^

We experimentally determined the ability of plants of producing seeds by self-fertilization during each season. To avoid undesirable side effects of local conditions, this experiment was performed in controlled conditions, in the experimental chambers, at the same time. We selected in March 2019, 10 plants growing in spring conditions and 24 plants growing in summer conditions at the onset of flowering. Each newly opened flower was marked and assigned to each of the following crosses: (1) self-pollination, where the flower was hand-pollinated with own pollen; (2) outcrossing, where the flower was pollinated with pollen from one different conspecific individual from the same season. Consequently, the number of flowers per treatment varied across individual, but was always higher than two, with a mean ± 1 s.e.m. equal to 6.1 ± 0.8 flowers per plant. Because all plants were located inside the experimental chambers, they were excluded from the visit of any pollinator. At the end of the fruiting period, we counted all flowers setting fruits, collected the fruits that were taken to the lab for counting their seeds as explained in the previous section.

### Spring and summer pollinators of *M. arvensis*

We identified the pollinators visiting the flowers of the studied populations both during spring and summer. For this, we conducted flower visitor counts in each population × season combination. We were able to obtain information in three populations (Malaha, Quesada, Tabernas). We visited each of these populations both during spring and during summer, always between 11:00 a.m. and 5:00 p.m. In these visits, we recorded the insects visiting the flowers of the plants for two hours without differentiating between individual plants. Each survey was done at least by two researchers simultaneously, sampling each population for at least 8–9 h person^−1^. We only recorded those insects contacting anthers or stigma and doing legitimate visits at least during part of their foraging at flowers. We did not record those insects only eating petals or thieving nectar without doing any legitimate visit. Previous studies using the same methodology carried out with similar Brassicaceae species and performing rarefaction analysis indicate that a sample of 130–150 insects provides an accurate estimate of the diversity of the pollinator assemblages^21,33,69,70^. To ensure that our sampling was representative, we recorded 471 insects in Malaha population, 334 insects in Quesada population and 300 insects in Tabernas population (Supplementary Table 13).

### Pollinators of the relative species

We compiled information on 308,096 insect visits belonging to 38 functional groups and more than 5000 morphospecies visiting 114 plant species belonging to the same tribe than *M. arvensis*, the Brassiceae (Supplementary Data 3). We use our data and data from the literature. In those species studied in the field (74 species), we conducted flower visitor counts in 1–16 populations per plant species. We visited the populations during the peak of the bloom, always at the same phenological stage and between 11:00 a.m. and 5:00 p.m. In these visits, we recorded the insects visiting the flowers of the plants for two hours without differentiating between individual plants. Insects were identified in the field, and some specimens were captured for further identification in the laboratory. We only recorded those insects contacting anthers or stigma and doing legitimate visits at least during part of their foraging at flowers. We did not record those insects only eating petals or thieving nectar without doing any legitimate visit. In addition, we included information on pollinators obtained from the literature to supplement our data (Supplementary Data 3 and 6). In this case, we use the information provided in the primary literature in terms of pollinator species and abundance at flowers. In this case, the plant species included in the network do not coexist, implying that this is a clade-oriented network rather than an ecological network^32^.

### Determination of pollination niches

In plant species with highly diverse pollination systems, like those included in this study, many pollinator species interact with the flowers in a similar manner, have similar effectiveness and exert similar selective pressures and are thus indistinguishable for the plant^10,33^. These pollinators are thus grouped into functional groups, that are the relevant interaction units in generalized systems^10,21,33,40^. We thereby grouped all pollinators visiting *M. arvensis* and the other Brassicaceae species using criteria of similarity in body length, proboscis length, morphological match with the flower, foraging behaviour, and feeding habits^21^. Supplementary Data 5 describes the 38 functional groups used in this study and Supplementary Data 4 shows the distribution of these functional groups among the studied Brassiceae taxa.

We determined the occurrence of different pollination niches in our studied populations and seasons using bipartite modularity, a complex-network metric. Modularity has proven to be a good proxy of interaction niches both in ecological networks, those included coexisting species or population, as well as in clade-oriented network, those including species with information coming from disparate and contrasting sources^32^. We constructed a weighted bipartite network, including pollinator data of four populations both during spring and summer flowering period. In this network, we pooled the data from the different individuals in a population and did not consider the time difference involved in sampling across different species. We removed all plant species with <20 visits. We subsequently determined the modularity level in this weighted bipartite network by using the QuanBiMo algorithm^71^. This method uses a simulated annealing Monte-Carlo approach to find the best division of populations into modules. A maximum of 10^10^ MCMC steps with a tolerance level = 10^−10^ were used in 100 iterations, retaining the iterations with the highest likelihood value as the optimal modular configuration. We tested whether our network was significantly more modular than random networks by running the same algorithm in 100 random networks, with the same linkage density as the empirical one^72^. Modularity significance was tested for each iteration by comparing the empirical versus the random modularity indices using a *z*-score test^71^. After testing the modularity of our network, we determined the number of modules^73^. We subsequently identified the pollinator functional groups defining each module and the plant species that were ascribed to each module. Modularity analysis was performed using R package bipartite 2.0^74^.

### Pollinator preference experiments

We carried out two experiments, one in late June 2019 and the other in late February/early March 2020, where 10 plants displaying spring-type flowers and 10 plants displaying summer-type flowers were offered at the same time to pollinators in a natural *M. arvensis* population. Plants were experimentally grown in chambers under each of the levels of the Treatment 2 conditions (spring conditions and hot summer conditions) and taken to the field in pots. Plants with each type of flowers were randomly distributed in a 5 × 4 grid with plants separated one metre. We performed ten 2-h trials in 2019 and nine 2-h trials in 2020, where the abundance and identity of the insects visiting the flowers of each experimental plant were recorded. Each trial was done by two researchers simultaneously, totalling 40 h of observation in 2019 and 36 h in 2020. Trials were performed between 11:00 and 13:00 local time in 2019 and between 12:00 and 14:00 local time in 2020. We counted each day the number of open flowers per plant, and, to control per between-plant differences in number of open flowers, insect abundance was expressed in number of visits per flower per hour. Afterwards, we followed the approach explained in the previous section to build up the bipartite interaction network, determine modularity and obtain the different modules, using only those plants having more than 10 visits per flower per hour (18 plants in the 2019 experiment and 20 plants in the 2020 experiment).

### De novo transcriptome analysis

In five plants from Treatment 1 (labelled MAR-70, MAR-81, MAR-83, MAR-98, and MAR-120), we sampled one starting-to-open flower bud during period 1 (experimental spring conditions) and another flower bud during period 2 (experimental mild summer conditions). We introduced the flower buds in liquid N_2_ and conserved them at −80 °C until RNA extraction. We used the RNeasy Plant Mini Kit (Qiagen) for total-RNA extraction. We checked the quality and quantity of the extracted RNA with a NanoDrop 2000 spectrophotometer (Thermo Fisher Scientific, Wilmington, DE, USA). The 10 RNA samples (five individuals, two conditions) were sequenced by Macrogen Inc. (Seoul, South Korea) after treatment with RiboZero to remove ribosomic RNA^75^. Libraries were produced using the TruSeq Stranded Total RNA LT Sample Prep Kit (Plant) and sequenced in an Illumina Novaseq6000 platform run (paired-end 150 bp) for a minimum of 40M reads/sample. See Supplementary Table 12 for information on the total number of bases reads and the total number of reads. RNA-Seq raw reads were submitted to Sequence Read Archive with the project accession number PRJNA604514. We retrieved 171,210 trinity genes (Supplementary Table 12 and Supplementary Data 1), but only 47,440 passed the criteria for inclusion in the analyses (at least 10 counts per million in spring or summer conditions).

We checked the quality of the raw sequences with FastQC^76^. After that, we used cutadapt (v. 1.15)^77^ and sickle (v. 1.33)^78^ to quality trim and remove adaptors. To produce a reference transcriptome, we concatenated the libraries (only paired sequences) and submitted them to RNA-Seq de novo assembly using Trinity v2.8.4^79^. A total of more than 372 Mbp were assembled in 424,981 transcripts (171,210 trinity ‘isogenes’), with a median contig length of 499 bp, an average length of 876.97 bp, and 10% of the assembled nucleotides appearing in transcripts of more than 4257 bp. We used trinity add-on scripts to compute contigs statistics with the help of Bowtie2 (v. 2.3.4.1)^80^. A total of 97.24% of reads aligned to the reference, with >80% aligned more than one time. We performed a BLASTX search of the assembled transcriptome to the SWISSPROT database and found that 10,696 proteins of this database mapped in more than 90% of their sequences.

### Analysis of DEG

We estimated the abundance of transcripts and genes using the Trinity abundance_estimates_to_matrix.pl script. Since the objective was to compare the within-individual expression of genes, we produced a matrix of raw gene counts as the input for the following analyses. We normalized (TMM)^81^ and filtered (conserving genes with at least 10 transcripts per gene and treatment) the matrix using edgeR^82^. A total of 47,870 genes passed this criterion. We analysed this matrix using a design of repeat samples with two conditions (spring as control and summer) and fit a negative binomial generalized log-linear model to the read counts for each gene. We selected as DEG the genes with false discovery rate adjusted-*P* values < 0.05.

### Annotation and GO-enrichment analysis

We used Trinotate v3^83^ to annotate the 47,870 genes using the uniport_sprot and Pfam-A databases, and mapping the longest isoform of each of those genes. Additionally, we used sma3s^84^ with similar results. For the gene ontology (GO) enrichment analyses, we used the Bioconductor package goseq^85^. As this package requires gene lengths, we obtained them using the Trinity script TPM_weighted_gene_length.py. We compared the GO terms of DEG with those of the 47,870 genes that passed the previous filtering.

### Statistical analyses of floral integration

Floral integration was computed as the relative variance of eigenvalues of the covariance matrix of the five floral traits included in this study^86^. One aspect of integration is that variation is concentrated in one or a few of the available dimensions^87^. As a consequence, there will be one or a few large and many small eigenvalues for the covariance matrix of integrated data, whereas eigenvalues of the covariance matrix will be more homogeneous for data lacking integration. To control for among-species differences in sampling size, we re-scaled the relative variance of eigenvalues by the total variance and number of dimensions^87^. By doing this, corolla shape integration ranges between 0 and 1 and can be interpreted as the percentage of integration regarding the maximum possible integration. This index is thus directly comparable to other integration indices found using different approaches.

### Statistical analyses of within-individual floral plasticity

The magnitude and significance of the within-individual plasticity were calculated for each floral and foliar trait by random slope mixed models, an analysis that fit individual-level reaction norms and thus assesses their variation in a single step^88^. We considered in all these models as explanatory variable the average daily temperature of each population or treatment during each season (spring and summer) or experimental condition (Supplementary Table 1). We first determined the overall population-level average effect of temperature on each response variable including temperature as a fixed effect function. Second, we quantified how much variation there is among individuals around the average population-level function by including in a second model the individual as a random effect (random intercepts). Finally, we quantified the variation around the average responses in the slopes of the individual reaction norms by adding a random regression term to the model. The temperature was included in all these models mean-centred^88^. Because we compared phenotypes between two environments, we always fitted linear models. The magnitude and significance of the population-level plasticity were found by estimating the coefficient of the fixed effect function. The among-individual differences in the magnitude of each trait were statistically tested by comparing the log-likelihoods of the first and second models and performing a likelihood ratio test (LRT)^88^. Similarly, the among-individual differences in the slopes of their reaction norms (the occurrence of G × E interaction) were statically tested by comparing the log-likelihoods of the second and third models and performing a LRT. The goodness-of-fit of these three models were compared by means of their Akaike Information Criteria (AIC)^88^.

### Bayesian generalized multivariate multilevel models

Plasticity integration was determined by quantifying the correlations between the plasticities of pair of traits. For this, we used Bayesian generalized multivariate multilevel models^89^. The structure of these models was similar to that explained previously for random regression, including individuals as random intercepts and slopes. As the dependent variable, we used a composite variable including all the five floral traits analysed independently in the previous analyses. We scaled and centred both the independent and dependent variables and used weakly informative prior with normal distribution and centred on zero^90^. We ran four chains with 2000 iteration each, burning 1000 samples per chain. In total, we analysed 400 post-warmup samples. We got in all cases the potential scale reduction factor on split chains to be 1 or very close to 1 at convergence. Significance was obtained for each effect by means of the posterior distribution of the 95% credible interval of its mean estimate. Bayesian analyses were performed in the R packages ‘brm’^89,91^.

### Reporting summary

Further information on research design is available in the Nature Research Reporting Summary linked to this article.

## Supplementary information

Supplementary Information

Peer Review File

Descriptions of Additional Supplementary Files

Supplementary Dataset 1

Supplementary Dataset 2

Supplementary Dataset 3

Supplementary Dataset 4

Supplementary Dataset 5

Supplementary Dataset 6

Reporting Summary

## Data Availability

Source data are provided with this paper. All raw sequence reads have been deposited in the NCBI SRA database under BioProject accession number PRJNA604514. Source data are provided with this paper.

## Source data

Source Data
