# Supplementary material for: Within-individual phenotypic plasticity in flowers fosters pollination niche shift
Source: Nat Commun. 2020 Aug 11;11:4019. doi: 10.1038/s41467-020-17875-1 (PMC7419554; doi:10.1038/s41467-020-17875-1)
Supplement: Supplementary file 1 — Supplementary Information [file 41467_2020_17875_MOESM1_ESM.pdf]

SUPPLEMENTARY FIGURES

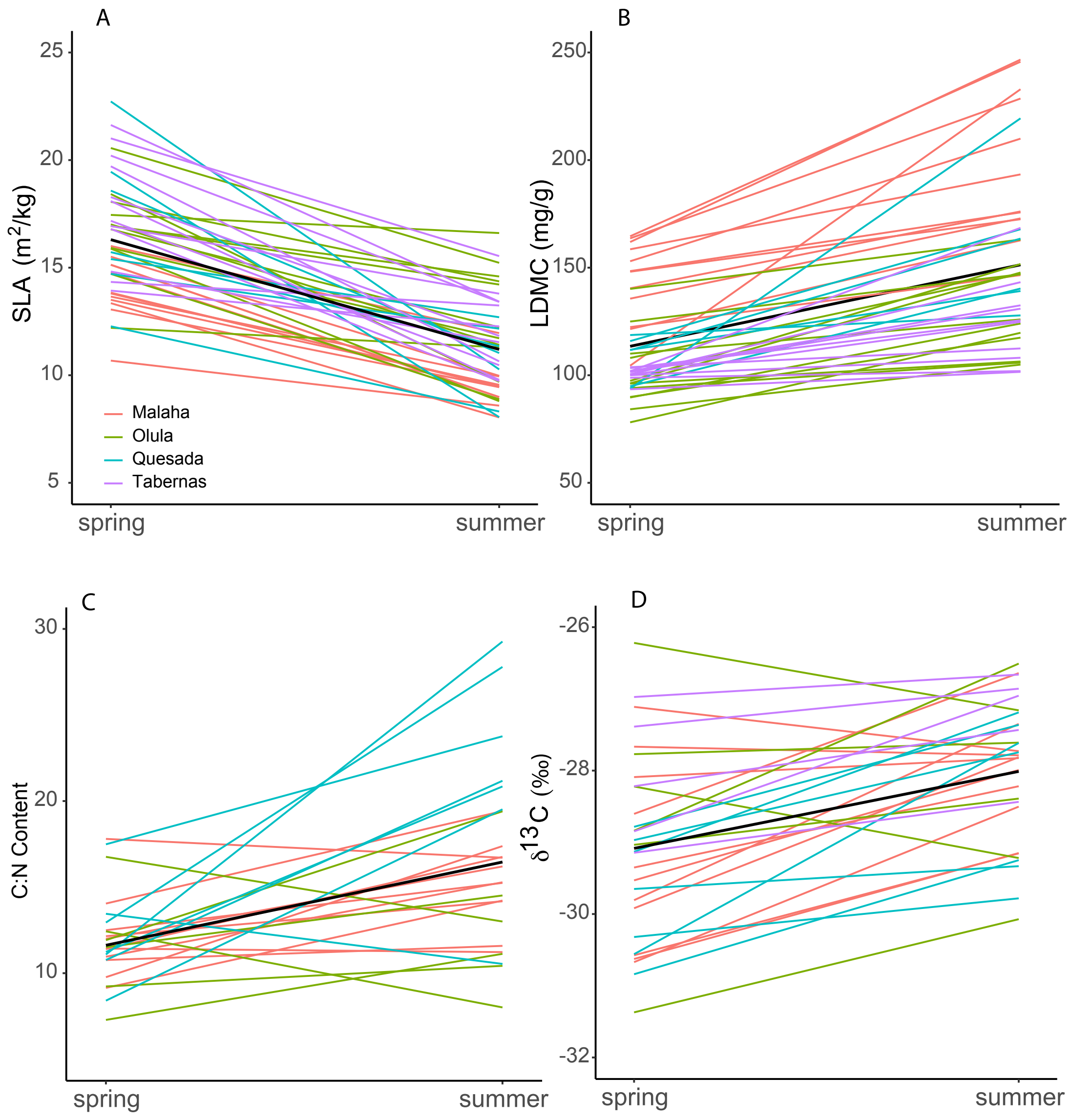

Supplementary Figure 1. Individual reaction norms of foliar traits. Individual reaction norms, pooling the individuals of the four studied populations (n = 36 individuals), of **A)** specific leaf area, in m^2 kg^{-1}; **B)** leaf dry matter content, in mg g^{-1}; **C)** Carbon to nitrogen relative content ratio; **D)** isotopic signature of C, $\delta^{13}C$, in ‰.

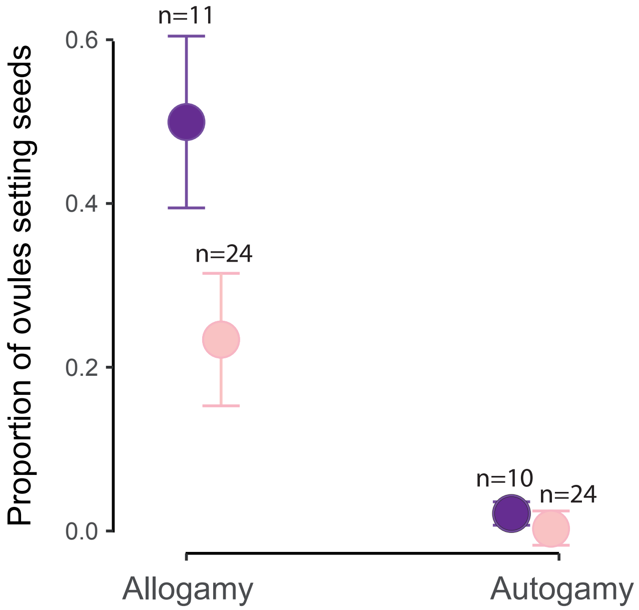

Supplementary Figure 2. Mating system of plastic flowers. Female fertility of the spring and summer flowers hand-pollinated with pollen with the same flower (autogamy) or with pollen from a different individual (allogamy). *n* is the number of individual plants per treatment. There were differences between seasons (z = 6.7, p<0.0001), and between pollination treatments (z = 4.4, p<0.0001), but not interaction (z = 1.0, p = 0.321, binomial GLM).

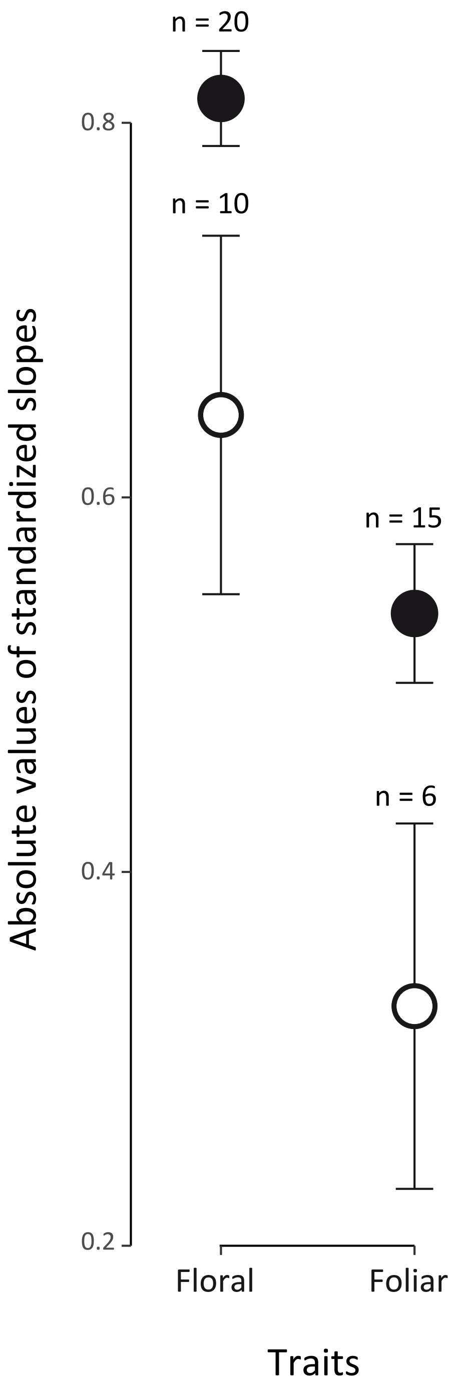

Supplementary Figure 3. Slope comparison. Mean population-level slopes (in absolute values) ± 1 s.e.m. for floral and foliar traits quantified in field (black dots) and experimental conditions (white dots). There was statistical differences between conditions ($F = 9.0$, $p = 0.004$) and type of traits ($F = 29.1$, $p < 0.0001$), but no their interaction ($F = 0.1$, $p = 0.727$, two-way ANOVA).

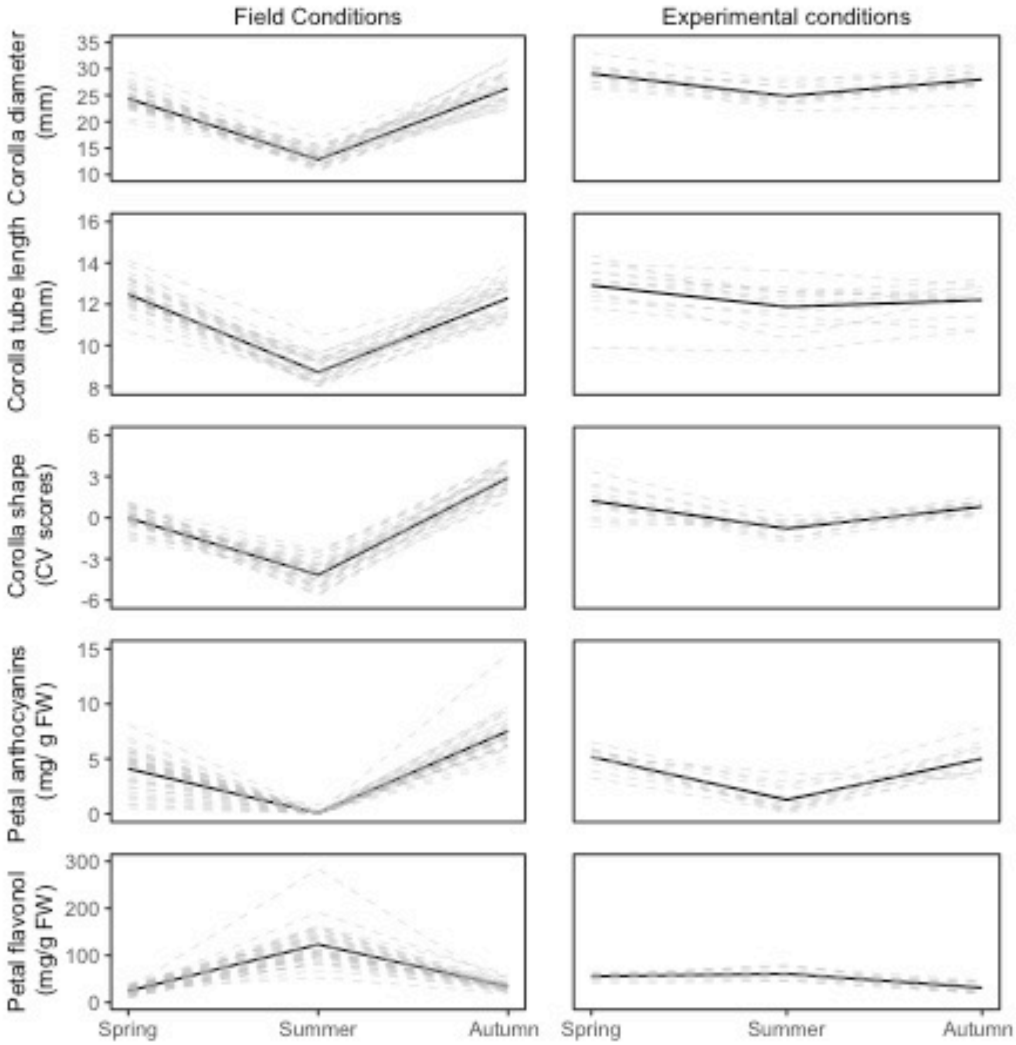

Supplementary Figure 4. Reversible individual reaction norms of floral traits in field and experimental conditions. Individual reaction norms of the five floral traits for those plants flowering during spring, summer and autumn both in field and experimental conditions. Reaction norms were linearly modelled as changed between each season subsequently.

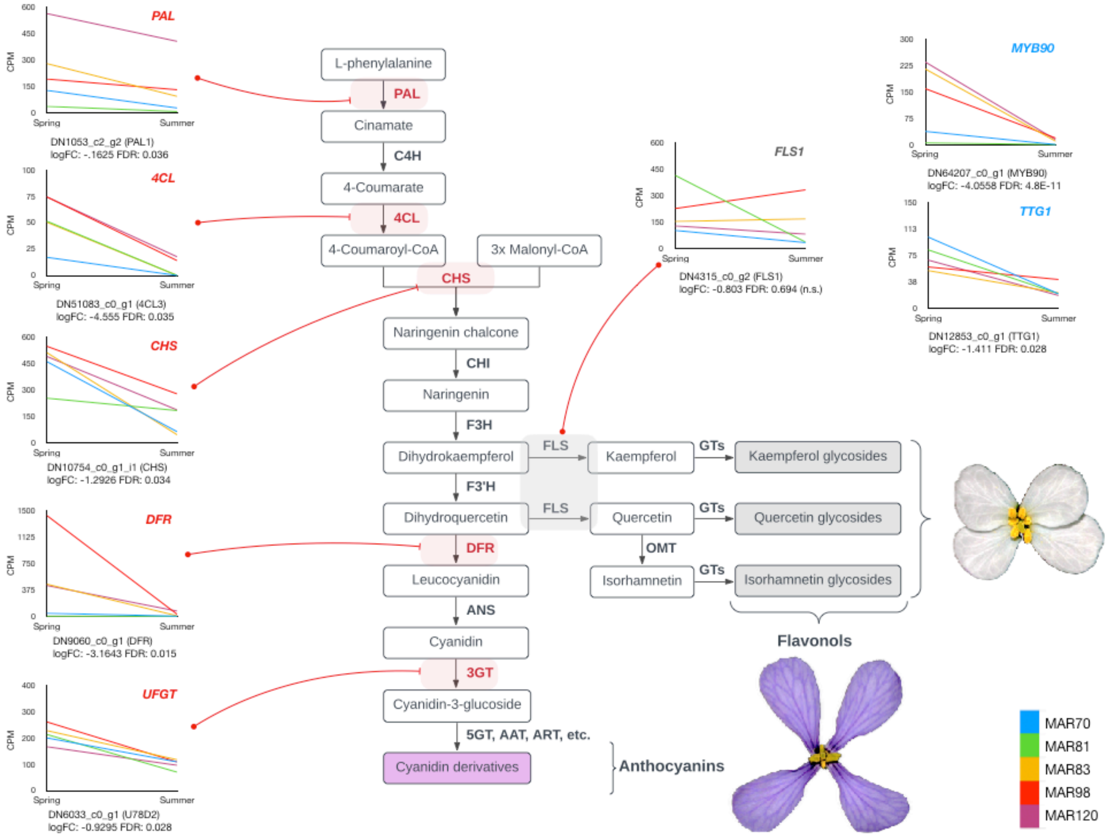

Supplementary Figure 5. Simplified anthocyanin biosynthetic pathway for spring and summer flowers of *Moricandia arvensis*. Enzymatic activities (capital letters next to arrows), gene expression patterns (line graphs at the side of each step) and metabolic products are indicated. Enzyme with relative significant and non-significant differential gene expression is highlighted in red and grey, respectively; whereas in blue is showed two regulatory genes with significant differential gene expression. Colour lines represent reaction norms of the five plants used in the transcriptomic study. Main anthocyanins and flavonols detected by UPLC–ESI–TOF–MS are in lilac and grey boxes, respectively (see Supplementary Table 1). The lilac colour of *M. arvensis* spring petals is produced by the accumulation of cyanidin derivatives, whereas *M. arvensis* summer petals only accumulate flavonols. PAL, phenylalanil ammonia-lyase; C4H, cinnamate 4-hydroxylase; 4CL, coumarate CoA ligase; CHS: chalcone synthase; CHI, chalcone isomerase; F3H, flavanone-3-hydroxylase; F3'H, flavonoid 3'hydroxylase; FLS, flavonol synthase; DFR, dihydroflavonol 4-reductase; ANS, anthocyanidin synthase; 3GT, flavonoid-3-O-glucosyltransferase; 5GT, flavonoid-5-O-glucosyltransferase; AAT, anthocyanin acyltransferase; RT, anthocyanin rhamnosyl transferase; GTs, glucosyltransferases; OMT, methyltransferase.

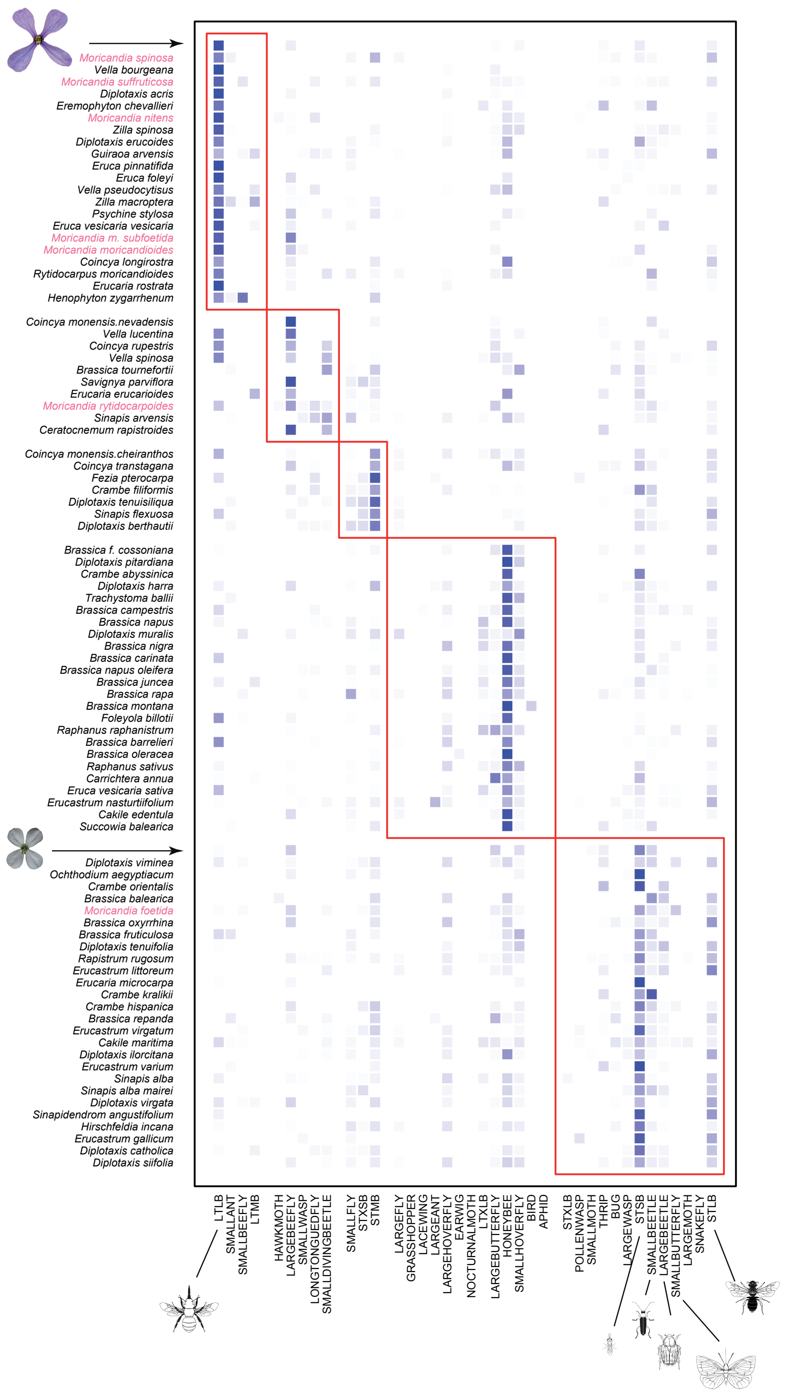

Supplementary Figure 6. Outcome of the modularity analysis performed on the bipartite weighted network built upon the visitation rate of the main 38 pollinator functional groups in 123 species belonging to the tribe Brassiceae. *Moricandia arvensis* was divided in those insects visiting spring flowers (represented by a spring-type flower) and those visiting summer flower (represented by a summer-type flower). We have highlighted the other *Moricandia* species and sketched those pollinator groups defining the two modules where *M. arvensis* is located. Insect silhouettes drawn by Divulgare (www.divulgare.net).

SUPPLEMENTARY TABLES

	Latitude	Longitude	# Plants marked/flowering Spring	Summer	Autumn	Average Daily Temperature Spring	Summer	Autumn
**Natural Populations**								
Tabernas	37° 00.3' N	2° 27.4' W	50	28	-	16.06	26.48	
Olula	37° 23.3' N	2° 17.9' W	50	27	-	15.08	26.69	
Quesada	37° 48.3' N	3° 03.4' W	50	22	-	15.54	27.92	
Malaha	37° 08.4' N	3° 43.9' W	50	40	36	15.09	26.82	17.07
**Experimental Conditions**						**Period 1**	**Period 2**	**Period 3**
Treatment 1			30	29		14.16	23.75	
Treatment 2			30	29		14.16	28.75	
Treatment 3			15	14		14.16	14.16	
Treatment 4				15	13	14.16	23.75	14.16

Supplementary Table 1. Average daily temperatures (°C) of each site, season and treatment included in this study. Natural population refers to the four populations studied in the SE Spain. Experimental Conditions refers to the different treatments carried on controlled conditions to test the effect of temperature and photoperiod on the triggering of floral plasticity (see methods for details). Autumn flowering was checked only in Malaha population

Site	std Slope	P values E	G	G x E	AIC Fixed term	Random Intercept	Random Slope
**Specific leaf area ($m^2\ kg^{-1}$)**							
Tabernas	-0.65	0.0001	0.4166	0.7534	174.2	175.6	179.0
Olula	-0.53	0.0001	0.0178	0.7041	183.1	179.5	182.8
Quesada	-0.66	0.0029	0.0518	0.0001	90.3	88.5	72.5
Malaha	-0.76	0.0001	0.0105	0.3889	133.9	128.5	130.6
**Leaf dry matter content (mg g-1)**							
Tabernas	0.59	0.0018	0.0337	0.0001	162.1	159.6	142.1
Olula	0.48	0.0001	0.0015	0.9278	186.5	178.5	182.3
Quesada	0.57	0.0127	0.2436	0.0001	94.0	94.7	79.0
Malaha	0.60	0.0001	0.0001	0.0041	169.9	157.5	150.5
**Carbon-to-Nitrogen ratio**							
Tabernas	-	-	-	-	-	-	-
Olula	0.27	0.2770	0.0001	0.0001	153.3	128.6	-321.4
Quesada	0.65	0.0046	0.0010	0.0001	91.6	82.8	-255.0
Malaha	0.54	0.0015	0.0001	0.0001	175.0	135.9	-348.4
**$\delta^{13}C$ (‰)**							
Tabernas	0.51	0.0377	0.0001	0.0001	73.2	45.9	-50.8
Olula	0.20	0.2027	0.0001	0.0001	154.1	98.6	-179.1
Quesada	0.55	0.0024	0.0001	0.0001	103.5	67.6	-125.6
Malaha	0.51	0.0006	0.0001	0.0001	177.0	125.8	-213.2

Supplementary Table 2. Phenotypic plasticity in functional and physiological traits. Outcome of the random regressions testing the change in foliar traits from spring to summer in the four studied populations. *Std Slope* is the population-wide among-season standardized slope of the individual reaction norms. *P-values* is the significance of the population-wide reaction-norm slope (E), the differences in trait values among individuals (G) and the among-individual differences in individual reaction-norm slopes. *AIC* is the increase in model fitting considering only the seasonal differences in plant trait (Fixed term), the differences among genotypes (Random intercept) and the interaction between season and genotype (Random slope).

Species	#	Species	#	Species	#
Brassica barrelieri	65	*Diplotaxis ilorcitana*	39	*Moricandia moricandioides*	132
Brassica fruticulosa	60	*Diplotaxis pitardiana*	25	*Moricandia nitens*	125
Brassica napus	61	*Diplotaxis siifolia*	65	*Moricandia spinosa*	81
Brassica nigra	64	*Diplotaxis tenuisiliqua*	65	*Moricandia suffruticosa*	130
Brassica oxyrrhina	179	*Diplotaxis viminea*	70	*Psychine stylosa*	140
Brassica repanda	99	*Diplotaxis virgata*	61	*Raffenaldia primuloides*	29
Brassica souliei	64	*Eremobium aegyptiacum*	49	*Raphanus pugioniformis*	43
Brassica tournefortii	59	*Eremophyton chevallieri*	72	*Raphanus raphanistrum*	135
Cakile maritima	73	*Eruca foleyi*	125	*Raphanus sativus*	68
Ceratocnemum rapistroides	66	*Eruca pinnatifida*	68	*Rapistrum rugosum*	76
Coincya longirostra	56	*Eruca vesicaria*	153	*Rytidocarpus moricandioides*	164
Coincya monensis cheiranthos	159	*Erucaria crassifolia*	50	*Savignya parviflora*	62
Coincya monensis nevadensis	39	*Erucaria erucarioides*	108	*Sinapis alba*	141
Coincya monensis orophila	30	*Erucastrum gallicum*	48	*Sinapis arvensis*	6
Coincya rupestris	57	*Erucastrum littoreum*	66	*Sinapis flexuosa*	60
Coincya transtagana	72	*Erucastrum nasturtiifolium*	126	*Vella aspera*	73
Crambe filiformis	119	*Erucastrum varium*	69	*Vella bourgeana*	83
Crambe hispanica	78	*Erucastrum virgatum*	161	*Vella lucentina*	67
Crambe kralikii	42	*Fezia pterocarpa*	54	*Vella pseudocytisus*	67
Diplotaxis berthautii	52	*Foleyola billotii*	65	*Vella spinosa*	53
Diplotaxis catholica	89	*Guiraoa arvensis*	70	*Zilla macroptera*	58
Diplotaxis erucoides	61	*Henophyton zygarrhenum*	22	*Zilla spinosa*	48
Diplotaxis harra	147	*Hirschfeldia incana*	68		
Diplotaxis ibicensis	67	*Moricandia foetida*	66		
Diplotaxis ollivieri	70	*Moricandia rytidocarpoides*	166		

Supplementary Table 3. Brassiceae species included in the geometric analysis to explore the corolla shape. # refers to the number of specimens analysed per species

Peak number	Flavonoid name (putative identification)[a]	RT (min)	Observed mass (*m/z*) [M+ H]+	Molecular formula [M+ H]+	Mass accuracy (PPM)	References previously reporting in Brassicaceae	Flavonoid (%)[c]	
							Liac petals	White petals
Anthocyanins								
A1	Cyanidin 3-xylosyl sinapoyl sophoroside-5-malonyl rutinoside[b]	2.55	1343.3486	C54H71O39	-6.4	1 (gd)	1.16	nd
A2	Cyanidin 3-caffeoyl feruloyl sophoroside-5-cinnamoyl rutinoside	2.61	1387.3762	C56H75O40	-5.3		0.75	nd
A3	Cyanidin 3-xylosyl sinapoyl sophoroside-5-malonyl sophoroside[b]	2.66	1359.3442	C57H67O38	-4.0	1 (gd)	1.40	nd
A4	Cyanidin 3-xylosyl sinapoyl glucoside-5-malonyl glucoside[b]	2.66	1035.2611	C46H51O27	8.5	1	1.13	nd
A5	Cyanidin 3-caffeoyl sinapoyl glucoside-5-malonyl rutinoside[b]	2.80	1197.2965	C55H57O30	11.3	2	1.09	nd
A6	Cyanidin 3-xylosyl sinapoyl glucoside-5-malonyl rhamnoside[b]	2.87	1019.2651	C46H51O25	0.0	1 (gd)	4.19	nd
A7	Cyanidin 3-caffeoyl feruloyl sophoroside-5-cinnamoyl rhamnoside	2.89	1241.3210	C50H65O36	8.6		2.41	nd
A8	Cyanidin 3-feruloyl sinapoyl xyloside-5-malonyl glucoside	2.97	1049.2776	C47H53O27	-0.2	2 (gd)	3.59	nd
A9	Cyanidin 3-ferulyl sinapoyl xyloxyl glucoside-5-malonyl glucoside	3.12	1211.3129	C56H59O30	2.9	2	1.50	nd
A10	Cyanidin 3-di-feruloyl sophoroside-5-cinnamoyl rhamnoside	3.21	1255.3403	C47H67O39	6.8		0.72	nd
Flavonols								
F1	Quercetin 3-galactoside-7-rhamnoside[b]	2.11	611.1583	C27H31O16	4.7	3	9.74	6.63
F2	Kaempferol 3-glucoside-7-sophoroside[b]	2.11	773.2137	C33H41O21	6.5	4 (gd)	10.59	1.15
F3	Kaempferol 3-rutinoside-7-glucoside[b]	2.14	757.2170	C33H41O20	-9.5	3	6.44	1.37
F4	Kaempferol 3-glucoside-7-rhamnoside[b]	2.19	595.1663	C27H31O15	-0.5	3	30.78	51.02
F5	Kaempferol 3-(xylosyl-rhamnosyl)-glucoside[b]	2.19	727.2088	C32H39O19	-8.9	3	2.04	4.15
F6	Isorhamnetin 3-rhamnoside-7-glucoside[b]	2.26	625.1559	C28H33O16	9.6	5	nd	2.19
F7	Kaempferol 3-p-coumaryl sophoroside-7-glucoside[b]	2.36	919.2493	C42H47O23	-1.6		1.08	2.73
F8	Kaempferol-3-glucosyl rutinoside-7-sophoroside	2.36	1081.2993	C48H57O28	-4.0		0.70	1.59
F9	Kaempferol 3-glucoside[b]	2.45	449.1073	C21H21O11	-2.4	6	2.95	4.61
F10	Kaempferol 3-caffeoyl glucosyl-1->4-rhamnoside[b]	2.50	757.1938	C36H37O18	-5.5	3	1.10	0.74

F11	Kaempferol 7-rhamnoside[b]	2.51	433.1113	C21H21O10	4.4	6	2.37	1.11
F12	Kaempferol 3-caffeoyl glucosyl-(1->2)-glucoside-7-cellobioside	2.56	1097.2776	C33H60O40	0.9	7	1.48	nd
F13	Kaempferol 3-sinapoyl-caffeoyl-sophoroside 7-glucoside[b]	2.61	1141.3063	C53H57O28	-9.6		0.50	nd
F14	Isorhamnetin 7-rhamnoside	2.65	463.1234	C22H23O11	-3.9	6	0.84	0.30
F15	Kaempferol 3-sinapoyl glucoside-7-glucosyl rhamnoside	2.85	963.2783	C44H51O24	1.3	1 (gd)	nd	0.70
F16	Isorhamnetin 3-feruloyl rhamnosyl-1->6-galactoside[b]	2.92	801.2231	C38H41O19	3.7		2.05	8.29
F17	6-Methoxykaempferol 3,7-bis (3-acetyl rhamnoside)[b]	2.95	963.2058	C32H37O17	-5.2		0.41	2.48
F18	8-Prenylkaempferol 3,7-diglucoside[b]	3.60	679.2247	C32H39O16	-5.6		5.40	6.68
F19	Sinocrassoside A9	3.60	841.2761	C38H49O21	-7.6	8	1.40	3.65
Isoflavones								
I1	Nigracin 4'-hydroxy-methylglutaryl-hexoside	2.77	649.1731	C30H33O16	7.5		1.50	nd
Hydroxycinnamic acids								
H1	1-Caffeoyl-5-feruloylquinic acid[b]	3.26	531.1522	C26H27O12	2.1		0.67	0.63

Supplementary Table 4. Flavonoid identification of petals of *Moricandia arvensis*. Results from the UPLC–ESI–TOF–MS biochemical analysis of purple and white petal extracts with flavonoid identifications. [a] Identification based on retention time, accurate mass and comparison with data from previously reported flavonoids for *Moricandia arvensis* and other Brassicaceae species. [b] Identification confirmed by molecular formula (see http://metabolomics.jp). [c] Relative peak intensities of each metabolite with respect to the sum of intensities of all flavonoids found in lilac and white petals. RT, retention time; gd, glucoside derivatives of flavonoids identified in these references.

Populations	std Slope	P values E	G	G x E	AIC Fixed term	Random Intercept	Random Slope
**Corolla diameter (mm)**							
Tabernas	-0.85	0.0001	0.0012	0.0595	219.1	210.7	209.0
Olula	-0.84	0.0001	0.0001	0.0017	178.0	158.3	149.6
Quesada	-0.85	0.0001	0.0006	0.0063	96.8	87.2	73.0
Malaha	-0.91	0.0001	0.0010	0.0288	146.1	137.3	133.6
**Corolla tube length (mm)**							
Tabernas	-0.65	0.0001	0.0317	0.0128	274.4	271.8	267.1
Olula	-0.75	0.0001	0.0001	0.0001	218.7	199.0	184.0
Quesada	-0.64	0.0001	0.0275	0.6857	196.4	193.5	196.8
Malaha	-0.89	0.0001	0.0001	0.0867	176.0	161.3	160.4
**Corolla shape (CV)**							
Tabernas	-0.99	0.0001	0.0026	0.7299	134.1	127.1	139.1
Olula	-0.91	0.0001	0.9999	0.0589	105.0	107.0	105.4
Quesada	-0.90	0.0001	0.1593	0.9999	254.7	250.7	254.7
Malaha	-0.93	0.0001	0.0127	0.0001	90.2	86.0	56.2
**Petal anthocyanins (cyanidin mg g^{-1} FW)**							
Tabernas	-0.90	0.0001	0.0001	0.0161	200.4	168.8	171.8
Olula	-0.63	0.0001	0.0001	0.0001	254.0	229.5	206.3
Quesada	-0.77	0.0001	0.0016	0.0052	150.0	142.0	135.5
Malaha	-0.83	0.0001	0.0001	0.0001	260.2	246.5	46.5
**Petal flavonols (kaempferol mg g^{-1} FW)**							
Tabernas	0.87	0.0001	0.2851	0.0001	215.6	216.5	196.0
Olula	0.73	0.0001	0.0022	0.0001	226.4	219.0	149.7
Quesada	0.59	0.0001	0.0377	0.0019	208.8	206.4	198.0
Malaha	0.83	0.0001	0.0001	0.0001	258.6	242.9	116.1

Supplementary Table 5. Phenotypic plasticity in floral traits in four populations from SE Spain. Outcome of the random regressions testing the change in floral traits from spring to summer in all populations. *Std Slope* is the population-wide among-season standardized slope of the individual reaction norms. *P-values* showed the significance of the population-wide reaction-norm slope (E), the differences in trait values among individuals (G) and the among-individual differences in their individual reaction-norm slopes. *AIC* refers to the increase in fitting of the models considering only the differences between season in plant trait (Fixed term), including the differences among genotypes (Random intercept) and the interaction between season and genotype (Random slope).

Floral traits	df	Model without site AIC	df	Model with site AIC	LRT	P-value
Corolla diameter	6	615.5	9	515.9	105.6	0.0001
Corolla tube length	6	834.8	9	786.8	54.0	0.0001
Corolla shape	6	373.5	9	275.6	103.9	0.0001
Petal anthocyanins	6	701.4	9	607.4	99.9	0.0001
Petal flavonols	6	575.0	9	538.1	42.9	0.0001

Supplementary Table 6. Among-site comparison in floral plasticity. It is shown the outcome of a Likelihood Ratio test comparing a model for each floral trait where site was included as random factor (N= 4 sites) against a model without this random factor.

Group-Level Effects	Estimate ± s.d.	Credible interval lower bound	Credible interval upper bound	Bulk Effective Sample Size	Tail Effective Sample Size
**Overall slopes of each trait pooling the four sites**					
Corolla diameter [R^2= 0.91 ± 0.01]	**-0.82 ± 0.02**	-0.87	-0.77	2357	2542
Corolla tube length [R^2= 0.82 ± 0.01]	**-0.74 ± 0.03**	-0.80	-0.68	2101	3212
Corolla shape [R^2= 0.92 ± 0.01]	**-0.91 ± 0.02**	-0.96	-0.87	2659	2976
Petal anthocyanins [R^2= 0.91 ± 0.01]	**-0.73 ± 0.04**	-0.81	-0.66	3404	2972
Petal flavonols [R^2= 0.91 ± 0.01]	**0.72 ± 0.04**	0.64	0.81	1235	1838
**Among – individual differences in slopes**					
Corolla diameter	**0.21 ± 0.02**	0.17	0.26	2220	3354
Corolla tube length	**0.28 ± 0.03**	0.22	0.34	1191	23335
Corolla shape	**0.22 ± 0.03**	0.18	0.26	2133	2939
Petal anthocyanins	**0.37 ± 0.03**	0.32	0.43	2211	2800
Petal flavonols	**0.42 ± 0.03**	0.37	0.37	2058	3288
**Between-trait correlation in individual slopes**					
Corolla diameter x Corolla tube length	**0.42 ± 0.11**	0.19	0.63	1730	2618
Corolla diameter x Corolla shape	**0.55 ± 0.10**	0.33	0.73	1321	2353
Corolla tube length x Corolla shape	0.05 ± 0.13	-0.20	0.31	2116	3012
Corolla diameter x Petal anthocyanins	-0.02 ± 0.11	-0.24	0.21	1756	2274
Corolla tube length x Petal anthocyanins	-0.04 ± 0.12	-0.27	0.19	1342	2319
Corolla shape x Petal anthocyanins	-0.12 ± 0.12	-0.35	0.10	1404	2210
Corolla diameter x Petal flavonols	**-0.37 ± 0.09**	-0.54	-0.17	2231	3102
Corolla tube length x Petal flavonols	**-0.54 ± 0.09**	-0.71	-0.34	1063	1840
Corolla shape x Petal flavonols	0.05 ± 0.10	-0.16	0.24	2820	3385
Petal anthocyanins x Petal flavonols	-0.01 ± 0.10	-0.21	0.17	3139	3482

Supplementary Table 7. Outcome of the Bayesian generalized multivariate multilevel models. It tests the effect of weighted temperature on the combined change in the five floral traits across the entire set of data (117 Individuals, 4 Sites). Significant differences occur when the credibility interval does not contain zero. It is shown the mean (estimate) and the standard deviation of the posterior distribution and its 95% Credible interval. The Effective Sample Size is an estimation of the number of independent samples from the posterior distribution that would be expected to yield the same standard error of the posterior mean as is obtained from the dependent samples returned by the MCMC algorithm. Among-groups differences are tested by comparing the standard deviations of the slopes of each trait between group levels. It is shown the proportion of the variance explained by the whole model for each individual trait as a Bayesian R^2.

Populations	std Slope	P values			AIC		
		E	G	G x E	Fixed term	Random Intercept	Random Slope
**Ovules**							
Tabernas	-0.42	0.0001	0.0001	0.0021	662.1	647.3	638.9
Olula	-0.55	0.0001	0.0001	0.0001	537.3	522.2	501.4
Quesada	-0.27	0.0001	0.0001	0.0001	1083.8	1025.3	993.8
Malaha	-0.43	0.0001	0.0001	0.0042	2109.6	1859.6	1852.6
**Seeds**							
Tabernas	-0.15	0.1061	0.0002	0.2209	687.1	675.4	676.4
Olula	0.12	0.1384	0.0001	0.1831	613.0	597.9	598.5
Quesada	-0.33	0.0002	0.0001	0.0001	1069.3	985.2	943.7
Malaha	-0.32	0.0001	0.0001	0.0001	2183.9	2072.9	2029.8

Supplementary Table 8. Phenotypic plasticity in seed set in four populations from SE Spain. See Extended Data Table 1 for legend.

Floral trait	std Slope	P values E	G	GxE	AIC Fixed term	Random Intercept	Random Slope
**Corolla diameter (mm)**							
Treatment 1	-0.74	0.0001	0.0011	0.2695	118.8	110.2	111.6
Treatment 2	-0.85	0.0001	0.0090	0.0254	93.8	89.0	85.7
Treatment 3	-0.53	0.0708	0.0021	0.0484	159.8	152.3	150.3
**Corolla tube length (mm)**							
Treatment 1	-0.37	0.0088	0.0001	0.0001	106.1	104.1	100.8
Treatment 2	-0.80	0.0001	0.0461	0.0256	166.3	149.7	134.1
Treatment 3	-0.39	0.0761	0.0001	0.1001	161.7	136.8	136.2
**Corolla shape (CV)**							
Treatment 1	-0.74	0.0001	0.9999	0.6129	127.2	129.2	132.3
Treatment 2	-0.85	0.0001	0.5852	0.2940	93.3	95.0	96.6
Treatment 3	-0.14	0.5907	0.0326	0.5501	163.6	161.0	163.8
**Petal anthocyanins (cyanidin mg g^{-1} FW)**							
Treatment 1	-0.88	0.0001	0.0025	0.0499	82.6	75.5	73.5
Treatment 2	-0.91	0.0001	0.0063	0.0001	64.7	59.2	-12.3
Treatment 3	0.57	0.0451	0.0001	0.0001	159.0	140.6	122.6
**Petal flavonols (kaempferol mg g^{-1} FW)**							
Treatment 1	0.09	0.6317	0.0004	0.2772	333.9	323.5	328.8
Treatment 2	0.21	0.3960	0.0178	0.0001	332.9	329.3	312.2
Treatment 3	-1.06	0.0003	0.0001	0.0041	144.9	127.3	123.1

Supplementary Table 9. Phenotypic plasticity in floral traits in experimental conditions. Treatment 1 refers to the pass from spring to mild summer conditions, Treatment 2 refers to the pass from spring to hot summer conditions. Treatment 3 refers to the pass from spring to spring conditions (Control). See Extended Table 1 for description of variables.

Populations	std Slope	P values E	G	GxE	AIC Fixed term	Random Intercept	Random Slope
**Corolla diameter (mm)**							
Field	0.77	0.0001	0.0001	0.0001	109.4	82.5	37.8
Experimental	0.62	0.0001	0.0001	0.0706	126.7	107.0	0.62
**Corolla tube length (mm)**							
Field	0.74	0.0001	0.0017	0.0033	172.7	164.9	157.4
Experimental	0.16	0.1758	0.0001	0.0267	151.1	131.6	128.3
**Corolla shape (CV)**							
Field	0.79	0.0001	0.0001	0.0698	414.3	375.0	373.6
Experimental	0.74	0.0001	0.9999	0.9727	112.1	114.1	118.0
**Petal anthocyanidin (cyanidin mg g^{-1} FW)**							
Field	0.78	0.0001	0.0021	0.0001	93.3	85.8	-25.8
Experimental	0.81	0.0001	0.2151	0.0001	95.6	96.1	79.9
**Petal flavonol (kaempferol mg g^{-1} FW)**							
Field	-0.67	0.0001	0.0001	0.0001	252.9	239.0	136.4
Experimental	-0.78	0.0001	0.0182	0.0565	104.4	100.8	104.3

Supplementary Table 10. Reversible phenotypic plasticity in floral traits. Outcome of the random regressions testing the change in floral traits from summer to autumn in Malaha population (Field) and in Treatment 4 (Experimental). Legend as Table S4.

Site	Trait values		
	**Spring**	**Summer**	**Autumn**
**Field Conditions**			
Corolla diameter (mm)	24.4±0.04	12.8±0.03	26.3±0.04
Corolla tube length (mm)	12.8±0.01	8.7±0.01	12.3±0.01
Corolla shape (CV)	0.01±0.01	-4.2±0.01	2.9±0.01
Petal anthocyanidin (cyanidin mg g^{-1} FW)	4.1±0.03	0.1±0.00	7.5±0.02
Petal flavonol (kaempferol mg g^{-1} FW)	24.2±0.13	123.1±0.6	33.6±0.14
**Experimental Conditions**			
Corolla diameter (mm)	26.7±0.11	20.8±0.10	25.2±0.11
Corolla tube length (mm)	12.9±0.04	11.7±0.04	12.2±0.03
Corolla shape (CV)	1.2±0.04	-0.7±0.01	0.8±0.03
Petal anthocyanidin (cyanidin mg g^{-1} FW)	5.2±0.04	1.3±0.05	5.0±0.06
Petal flavonol (kaempferol mg g^{-1} FW)	55.3±0.4	60.8±0.5	31.1±0.4

Supplementary Table 11. Reversible phenotypic plasticity in floral traits. Field conditions refers to plants from Malaha population. Experimental conditions refer to plants from treatment 4.

	spring		summer	
**Individual**	**Total read bases (bp)**	**Total reads**	**Total read bases (bp)**	**Total reads**
70	7,192,526,828	47,632,628	7,183,531,456	47,573,056
81	7,624,189,320	50,491,320	6,574,315,010	43,538,510
83	7,438,282,348	49,260,148	6,352,743,952	42,071,152
98	7,713,896,004	51,085,404	7,808,254,696	51,710,296
120	6,576,313,344	43,551,744	7,103,341,698	47,041,998

Supplementary Table 12. Total read bases and total number of reads for the sequenced samples of each of the five individuals, one sample taken in experimental spring conditions and the other take in experimental mild summer conditions.

Site	LTLB	STLB	STMB	STSB	Beetle	Fly	Hoverfly	Beefly	Butterfly	Moth	Thrip
**SPRING**											
Malaha	175	0	0	4	0	0	2	0	2	0	0
Quesada	190	0	0	0	0	0	4	0	0	0	0
Tabernas	160	6	0	20	0	2	2	50	0	0	0
**SUMMER**											
Malaha	5	3	0	127	10	0	44	22	66	7	4
Quesada	0	0	0	80	10	0	0	40	0	0	10
Tabernas	0	0	0	0	40	0	0	10	0	0	10

Supplementary Table 13. Frequency of occurrence of each pollination functional group in each plant population during spring and summer. LTLB: Long-tongued large bees, STLB: short-tongued large bees, STMB: Short-tongued medium-sized bees, STSB: short-tongued small bees.

SUPPLEMENTARY REFERENCES

1. Tatsuzawa, F., Aiba, Y., Morino, T., Saito, N., Shinoda, K., Kato, K., & Honda, T. Copigmentation with acylated anthocyanin and kaempferol glycosides in violet and purple flower cultivars of *Aubrieta×cultorum* (Brassicaceae). J. Jap. Soc. Hort. Sci. **81**, 275–284 (2012).
2. Saito, N., Tatsuzawa, F., Nishiyama, A., Yokoi, M., Shigihara, A., & Honda, T. Acylated cyanidin 3-sambubioside-5-glucosides in *Matthiola incana*. Phytochemistry **38**, 1027–1032 (1995).
3. Marrelli, M., Morrone, F., Argentieri, M., Gambacorta, L., Conforti, F., & Avato, P. Phytochemical and biological profile of *Moricandia arvensis* (L.) DC.: an inhibitor of pancreatic lipase. Molecules **23**, 2829 (2018).
4. Berreghioua, A.; Cheriti, A. Phytochemical investigation of the medicinal plant *Moricandia arvensis* L. from Algerian Sahara. Asian J. Pharm. Clin. Res. **5**, 450–453 (2018).
5. Saito, K., Yonekura-Sakakibara, K., Nakabayashi, R., Higashi, Y., Yamazaki, M., Tohge, T., & Fernie, A. R. The flavonoid biosynthetic pathway in *Arabidopsis*: structural and genetic diversity. Plant Physiol. Biochem. **72**, 21–34 (2013).
6. Yonekura-Sakakibara, K., Tohge, T., Matsuda, F., Nakabayashi, R., Takayama, H., Niida, R., Watanabe-Takahashi, A., Inoue, E., & Saito, K. Comprehensive flavonol profiling and transcriptome coexpression analysis leading to decoding gene–metabolite correlations in *Arabidopsis*. Plant Cell **20**, 2160–2176 (2008).
7. Nielsen, J. K., Nørbæk, R., & Olsen, C. E. Kaempferol tetraglucosides from cabbage leaves. Phytochemistry **49**, 2171–2176 (1998).
8. Buckingham, J. & Munasinghe, V. R. N. Dictionary of Flavonoids, CRC Press, Florida, USA (2015).
