# Supplementary material for: Within-individual phenotypic plasticity in flowers fosters pollination niche shift
Source: Nat Commun. 2020 Aug 11;11:4019. doi: 10.1038/s41467-020-17875-1 (PMC7419554; doi:10.1038/s41467-020-17875-1)
Supplement: Supplementary file 3 — Descriptions of Additional Supplementary Files [file 41467_2020_17875_MOESM3_ESM.pdf]

Descriptions of Additional Supplementary Files

Supplementary Data 1.

Description: Significantly Differential Expressed Genes (DEGs) sensu Trinity, with the logFold-Change (positive values indicate overexpression in summer), FDR adjusted P-values, and annotation with trinotate and Sma3s.

Supplementary Data 2.

Description: Enrichment analysis of GO terms of the differential expressed genes. Over-represented (enriched) terms are indicated with + and under-represented (depleted) with –. BP= biological process; CC= cellular component; MF= molecular function.

Supplementary Data 3.

Description: Number of Brassiceae species studied both in the field (in bold) and using data from the literature.

Supplementary Data 4.

Description: Frequency of occurrence of each pollination functional group in each Brassiceae species. LTLB: Long-tongued large bees, STLB: short-tongued large bees, LTMB: Long-tongued medium-sized bees, LTXLB: Long-tongued extra-large bees, STSB: short-tongued small bees, STMB: shorttongued medium-sized bees, STLB: short-tongued large bees, STXLB: short-tongued extra-large bees, STXSB: short-tongued extra-small bees.

Supplementary Data 5.

Description: Pollinator functional groups. Brief description of the functional groups of the insects visiting the flowers of the studied species.

Supplementary Data 6.

Description: Pollinator Database. Databse of the insects visiting the flowers of Brassiceae species. Both own data and data from the literature are included.
