# Supplementary material for: Within-individual phenotypic plasticity in flowers fosters pollination niche shift
Source: Nat Commun. 2020 Aug 11;11:4019. doi: 10.1038/s41467-020-17875-1 (PMC7419554; doi:10.1038/s41467-020-17875-1)
Supplement: Supplementary file 10 — Reporting Summary [file 41467_2020_17875_MOESM10_ESM.pdf]

nature research

Corresponding author(s): José María Gómez

Last updated by author(s): 14/07/2020

Reporting Summary

Nature Research wishes to improve the reproducibility of the work that we publish. This form provides structure for consistency and transparency in reporting. For further information on Nature Research policies, see Authors & Referees and the Editorial Policy Checklist .

Statistics

For all statistical analyses, confirm that the following items are present in the figure legend, table legend, main text, or Methods section.

n/a	Confirmed	
☐	☒	The exact sample size (*n*) for each experimental group/condition, given as a discrete number and unit of measurement
☐	☒	A statement on whether measurements were taken from distinct samples or whether the same sample was measured repeatedly
☐	☒	The statistical test(s) used AND whether they are one- or two-sided<br>*Only common tests should be described solely by name; describe more complex techniques in the Methods section.*
☐	☒	A description of all covariates tested
☐	☒	A description of any assumptions or corrections, such as tests of normality and adjustment for multiple comparisons
☐	☒	A full description of the statistical parameters including central tendency (e.g. means) or other basic estimates (e.g. regression coefficient) AND variation (e.g. standard deviation) or associated estimates of uncertainty (e.g. confidence intervals)
☐	☒	For null hypothesis testing, the test statistic (e.g. *F*, *t*, *r*) with confidence intervals, effect sizes, degrees of freedom and *P* value noted<br>*Give P values as exact values whenever suitable.*
☒	☐	For Bayesian analysis, information on the choice of priors and Markov chain Monte Carlo settings
☐	☒	For hierarchical and complex designs, identification of the appropriate level for tests and full reporting of outcomes
☒	☐	Estimates of effect sizes (e.g. Cohen's *d*, Pearson's *r*), indicating how they were calculated

Our web collection on statistics for biologists contains articles on many of the points above.

Software and code

Policy information about availability of computer code

Data collection	NA
Data analysis	R version 3.5.0

For manuscripts utilizing custom algorithms or software that are central to the research but not yet described in published literature, software must be made available to editors/reviewers. We strongly encourage code deposition in a community repository (e.g. GitHub). See the Nature Research guidelines for submitting code & software for further information.

Data

Policy information about availability of data

All manuscripts must include a data availability statement. This statement should provide the following information, where applicable:

- Accession codes, unique identifiers, or web links for publicly available datasets
- A list of figures that have associated raw data
- A description of any restrictions on data availability

The data that support the findings of this study are available from the corresponding author upon reasonable request. Genetic data have been sent to GenBank SRA Archives

Field-specific reporting

Please select the one below that is the best fit for your research. If you are not sure, read the appropriate sections before making your selection.

☐ Life sciences ☐ Behavioural & social sciences ☒ Ecological, evolutionary & environmental sciences

Ecological, evolutionary & environmental sciences study design

All studies must disclose on these points even when the disclosure is negative.

Study description	Here we demonstrate the occurrence of discrete, reversible within-individual floral plasticity that modulates the interaction with pollinators. Individuals of Moricandia arvensis produce two types of flowers, large, cross-shaped, and UV-reflecting lilac in spring; small, rounded, and UV-absorbing white in summer. Transcriptional responses to changes in temperature and photoperiod mediated this plasticity. Plastic change in photosynthesis metabolism, from C2 in spring to values closer to C4 in summer, probably facilitates plants to bloom during summer and allow the expression of the floral plastic phenotype. By displaying two different but functionally and phenotypically integrated flowers, the same individuals interacted with different pollinator networks. The discrete floral plasticity described in this study allows M. arvensis to jump to a different region of the floral phenotypic space and exploit alternative pollination niches. We presume that phenotypic plasticity is an important but neglected factor facilitating shifts in biotic niches.
Research sample	We worked with Moricandia arvensis (Brassicaceae) a weedy herb from the Mediterranean area that inhabits the dry lands of Iberian Peninsula and North Africa. We chose this plant species because it flowers from February to July and displays two different types of flowers, the main focus of our study. We worked in a extense area of the SE Iberian Peninsula, a place where this plant species lives both associated to humans and also in natural ecosystems.
Sampling strategy	Sample size, both between and within plant, was determined by running GLMMs with individual as random factors. Number of plants per population (4 populations) was fixed to 50 to ensure enough statistical power. Plant ecological and physiological traits were replicated for each single plant. Insect surverys were performed every day of 2019 to ensure that any seasonal change in insect availability was captured. We did a field experiment in summer 2019 and a second one early spring 2020.
Data collection	Data were collected by all authors and by four technicians, always under our supervision, directly from the experimental plants
Timing and spatial scale	We started compiling data in January 2018 and ended in March 2020
Data exclusions	We excluded from our analyses those plants failing to flower during each season.
Reproducibility	All methods are exhaustively described in the Method section to ensure any researcher understands and can reproduce our study.
Randomization	In all experiments, plants were randomly allocated to each treatment. In field study, plants of similar size were chosen from large populations.
Blinding	NA

Did the study involve field work? ☒ Yes ☐ No

Field work, collection and transport

Field conditions	Dry lands of the Southeastern Iberian Peninsula, mostly badlands and semideserts
Location	Four populations in Andalusia region in Spain, belonging to Granada, Jaen, Almería provinces ranfing between 300 and 1200 m a.s.l. Geographical coordinate are provided in Supplementary Material. Climate is Mediterranean, with average daily temperature ranging from 15ºC in spring to 28ºC in summer.
Access and import/export	We have been extremely careful with not interfering with the natural dynamics of the ecosystems. Our study plant is a non-protected weed, so, no specific permit was required to study it. Because all the study was performed outside any protected area, no permit was either required.
Disturbance	NA

Reporting for specific materials, systems and methods

We require information from authors about some types of materials, experimental systems and methods used in many studies. Here, indicate whether each material, system or method listed is relevant to your study. If you are not sure if a list item applies to your research, read the appropriate section before selecting a response.

Materials & experimental systems

n/a	Involved in the study
☒	☐ Antibodies
☒	☐ Eukaryotic cell lines
☒	☐ Palaeontology
☒	☐ Animals and other organisms
☒	☐ Human research participants
☒	☐ Clinical data

Methods

n/a	Involved in the study
☒	☐ ChIP-seq
☒	☐ Flow cytometry
☒	☐ MRI-based neuroimaging
