# Supplementary material for: Within-individual phenotypic plasticity in flowers fosters pollination niche shift
Source: Nat Commun. 2020 Aug 11;11:4019. doi: 10.1038/s41467-020-17875-1 (PMC7419554; doi:10.1038/s41467-020-17875-1)
Supplement: Supplementary file 2 — Peer Review File [file 41467_2020_17875_MOESM2_ESM.pdf]

This manuscript has been previously reviewed at another journal that is not operating a transparent peer review scheme. The manuscript was considered suitable for publication without further review at *Nature Communications.*
